# Macrophage-to-myofibroblast transition-derived itaconate promotes bone metastasis in lung cancer through targeting of HSPA8

**DOI:** 10.1038/s12276-026-01799-9

**Published:** 2026-08-05

**Authors:** Jin Qian, Zhidan Tan, Jinfeng Wang, Tiantian Wei, Chongquan Huang, Shi Cheng, Guoqing Zhong, Huizhen Zou, Xia Kang, Linchong Sun, Yu Zhang

**Affiliations:** 1 Department of Orthopedics, Guangdong Provincial People’s Hospital (Guangdong Academy of Medical Sciences), Southern Medical University, Guangzhou, Guangdong China; 2 Engineering Technology Research Center of Functional Repair of Bone Defects and Biomaterials, Guangzhou, Guangdong China; 3 School of Medicine South China University of Technology, Guangdong Provincial People’s Hospital (Guangdong Academy of Medical Sciences), Southern Medical University, Guangzhou, Guangdong China; 4 Department of Musculoskeletal Oncology, Affiliated Cancer Hospital of Zhengzhou University, Henan Cancer Hospital, Zhengzhou, Henan China; 5 Tissue Stress Injury and Functional Repair Key Laboratory of Sichuan Province & Preclinical Research Center, General Hospital of Western Theater Command, Chengdu, Sichuan China; 6 College of Medicine, Southwest Jiaotong University, Chengdu, Sichuan China; 7 Medical Research Institute, Guangdong Provincial People’s Hospital, Guangdong Academy of Medical Sciences, Southern Medical University, Guangzhou, Guangdong China; 8 Guangdong Provincial Key Lab of Translational Medicine in Lung Cancer, Guangdong Provincial People’s Hospital, Guangdong Academy of Medical Sciences, Southern Medical University, Guangzhou, Guangdong China

**Keywords:** Bone cancer, Cancer therapeutic resistance, Immunoediting

## Abstract

Lung cancer bone metastasis carries a poor prognosis, yet the metabolic determinants driving tumour–stroma crosstalk remain largely elusive. Despite extensive investigations into itaconate, a prominent immunometabolite implicated in macrophage polarization, the precise mechanisms by which it modulates bone metastasis remain unresolved. Integrating metabolomics and transcriptomics profiling, the molecular landscape of lung cancer bone metastasis is delineated and the mechanistic role of itaconate is uncovered. Further ubiquitination proteomics of tumour cells and CRISPR–Cas9-mediated knockout of the gene encoding immune-responsive gene 1 (*IRG1*) confirmed the results in an animal model of lung cancer bone metastasis. The macrophage-to-myofibroblast transition generated cancer-associated fibroblasts that secreted elevated levels of itaconate, significantly accelerating tumour growth. A drug affinity responsive target stability screening pinpointed heat shock protein family A member 8 (HSPA8) as a direct molecular target of itaconate. Mechanistically, itaconate promoted HSPA8 ubiquitination and subsequent proteasomal degradation, thereby releasing activated transcription factor 4 (ATF4) from cytosolic sequestration. Liberated ATF4 translocated to the nucleus, where it bound the promoter region of phosphoserine aminotransferase 1 (PSAT1) to upregulate its expression. In vivo validation demonstrated that administration of adeno-associated virus-delivered PSAT1 short hairpin RNA or of a cell-penetrating itaconate antagonist significantly reduced tumour burden and prolonged survival. Our findings elucidate an unappreciated metabolic reprogramming axis in lung cancer bone metastases: macrophage-to-myofibroblast transition-derived itaconate alleviated cytoplasmic sequestration of ATF4 via HSPA8 ubiquitination, thereby activating its transcriptional target PSAT1. This mechanism converts immunometabolic byproducts into pro-tumorigenic signals that enhance bone metastasis. Notably, the HSPA8–ATF4–PSAT1 axis was identified as a key regulatory pathway governing metabolic reprogramming, thereby establishing a translational framework for targeting immunometabolic crosstalk in bone metastasis therapy.

## HSPA8–ATF4–PSAT1 axis fuels lung cancer bone metastasis

Lung cancer, a leading cause of cancer-related deaths, often leads to bone metastases, significantly impacting patient survival and quality of life. This study explores the cellular mechanisms driving metabolic reprogramming in bone metastases, focusing on the macrophage-to-myofibroblast transition as a key player. The researchers hypothesized that macrophage-to-myofibroblast transition cells secrete itaconate, a metabolite that reprogrammes tumour cell metabolism, promoting metastasis. Using patient samples and animal models, they identified itaconate as a central regulator, enhancing tumour proliferation by upregulating PSAT1, a serine synthesis enzyme. The study found that itaconate induces heat shock protein family A member 8 (HSPA8) degradation, releasing activated transcription factor 4 (ATF4), which upregulates phosphoserine aminotransferase 1 (PSAT1), driving tumour growth. These findings suggest that targeting the itaconate–HSPA8–ATF4–PSAT1 axis could offer new therapeutic strategies for lung cancer bone metastases, potentially improving patient outcomes. Future research should explore this axis in clinical settings to develop precise treatments. This summary was initially drafted using artificial intelligence, then revised and fact-checked by the author.

## Introduction

Lung cancer is the main cause of cancer-related death, and 30–40% of patients with lung cancer experience bone metastases during the disease process, and bone metastasis occurs in 30–40% of patients during the course of lung cancer, and up to 60% of patients already have bone metastasis at the time of initial diagnosis, significantly reducing overall survival and quality of life^1,2^. Bone metastasis of lung cancer remains incurable once established, and the cellular driver that predominantly reprogrammes the metabolic environment of the osseous niche is still undefined^3^. Here, the fundamental question of which population initiates and sustains this metabolic reprogramming is addressed. It is hypothesized that the macrophage-to-myofibroblast transition (MMT) constitutes a primary source of secreted metabolites within bone lesions and directly imposes a proliferative metabolic programme on tumour cells, thereby fuelling metastatic progression. Accordingly, this study systematically delineates the spatiotemporal dynamics of MMT during the osseous colonization of lung cancer, identifying the bioactive metabolite within the MMT secretome that is closely related to tumours, elucidating the molecular circuitry through which tumour cells sense and convert this metabolite into a growth signal, and ultimately evaluating whether interrupting this metabolic axis can halt bone metastases. The overarching goal is to establish a metabolic–immune combined therapeutic paradigm for lung cancer bone metastases.

Current treatments have achieved significant efficacy for lung cancer but have limited benefits for patients with bone metastases^4,5^. The obvious difference in the curative effect is mainly due to the special bone microenvironment^6^. Recent studies have consolidated the concept of cancer-associated fibroblasts (CAFs), which coevolve with disseminated tumour cells and coordinate therapeutic resistance across multiple solid tumours, including breast and pancreatic cancer^7,8^. However, the cellular ontogeny of CAFs remains contentious. Emerging evidence now positions the macrophage-to-myofibroblast transition as a hitherto underappreciated mechanism that generates functionally specialized CAFs within the metastatic bone microenvironment^9,10^. While MMT has been documented in renal scarring and oral squamous cell carcinoma^11,12^, its contribution to lung cancer bone colonization and therapeutic sensitivity has scarcely been explored.

In recent years, immune metabolites have emerged as a major research topic because of their ability to both directly affect tumour cells and control immune cell function^13–15^. Itaconate, an α,β-unsaturated carboxylic acid, is catalysed by mitochondrial aconitate decarboxylase 1 (also known as immune-responsive gene 1 (IRG1))^16^. Numerous studies have demonstrated its antibacterial and antioxidant properties in the macrophage anti-inflammatory response^17,18^. However, the source, concentration, target and role of itaconate in MMT are still largely unknown. Itaconate expression was higher in macrophages from tumours such as ovarian cancer and melanoma than in those from non-tumour tissue. The related catalytic enzyme, IRG1, was similarly preferentially overexpressed, while it was rarely found in normal tissues^19^. In non-small-cell lung cancer (NSCLC) and renal clear cell carcinoma, itaconate expression in tumour tissues was positively correlated with that of the transporter solute carrier family 13 member 3 (SLC13A3), and the prognosis of patients with high levels of itaconate was significantly worse^20^. In addition to lowering the 5-hydroxymethylation level of the tumour suppressor genes *p21* and *PTEN* (encoding phosphatase and tensin homologue) by blocking *TET2* (encoding ten-eleven translocation 2) activity, itaconate can also directly alkylate the PD-L1 CYS272 site to prevent its ubiquitination degradation, maintaining PD-L1 protein stability and promoting immune escape. In vitro and in vivo, blocking *IRG1* can dramatically reduce the proliferation of cancers^21^. Hence, metabolic reprogramming is increasingly considered to be a driver of macrophages. Itaconate, a metabolite derived from the tricarboxylic acid (TCA) cycle, is a central regulator that inhibits inflammatory flow while promoting immunosuppressive and repair-promoting phenotypes. However, whether and how this itaconate-centred circuit is retained, amplified or even reused after MMT is still completely unknown.

In this study, MMT was found to be the key driving mechanism of bone metastases in lung cancer. MMT directly acts on tumour cells by secreting itaconate, inducing metabolic reprogramming and accelerating tumour progression. Mechanistically, itaconate upregulated the expression of phosphoserine aminotransferase 1 (PSAT1), the rate-limiting enzyme of serine synthesis, with heat shock protein family A member 8 (HSPA8) as the direct target. The itaconate–HSPA8 axis upregulates PSAT1 expression by activating the activating transcription factor 4 (ATF4) signalling pathway, thus promoting the proliferation of cancer cells. Functional rescue experiments revealed that adeno-associated virus (AAV)-mediated *PSAT1* knockdown could significantly delay tumour progression, which confirmed that PSAT1 was a targeted effector of the MMT itaconate axis downstream. Therefore, in this study, the molecular mechanism through which itaconate induces the ubiquitination and degradation of HSPA8 was systematically analysed, the effects of its activation on ATF4 nuclear translocation and PSAT1 transcription were verified, and its ability to promote tumour proliferation and bone metastasis was clarified. These findings demonstrate a novel antitumour approach that targets immune metabolites, chaperones and metabolic enzymes.

## Materials and methods

### Sample collection and patient characteristics

Bone metastasis samples were collected from 82 patients with pathologically confirmed NSCLC bone metastases for histochemical analysis, following approval from the Ethics Committee of Guangdong Provincial People’s Hospital (2019-550 A). Written informed consent was obtained from all participants. The median age of the 82 patients was of 63 years, with a range from 37 to 82 years. Detailed clinical and molecular information, including age, sex, smoking history, histological subtype, metastasis site, αSMA expression and overall survival, is summarized in Supplementary Table 1.

### Animal experiments

All animal experiments and procedures were approved by the Institutional Animal Care and Use Committee at the Guangdong Provincial People’s Hospital. Male C57BL/6 and nude mice aged 6–8 weeks were used for experiments. To establish the bone metastasis model, 5 × 10^5^ A549 cells mixed with 1 × 10^6^ bone marrow-derived macrophages (BMDMs) or MMT cells (cells that have undergone MMT) were injected into the tibia following a previously described standard method^22^. In vivo experiments, 5 μg single-cell RNA or small interfering RNA (siRNA) were mixed with a transfection reagent and were then injected into the tibia in two separate injections of 5 μg each (totaling 10 μg per tibia), with the second injection administered 5 days later to maintain effective gene silencing and prolong the therapeutic effect, as siRNA is metabolized relatively quickly. 4-Octyl itaconate (4-OI; 12.5 mg/kg) was intraperitoneally (i.p.) injected once after the bone metastasis model had been established for 7 days; the same volume of dimethyl sulfoxide (DMSO) was used as a control. Bioluminescence images, histochemical analysis and micro-CT analysis were conducted 2 weeks after injecting A549 cells. The tumour-inoculated mice were sacrificed ~3 weeks later. This study was approved by the Institutional Animal Care and Use Committee of Guangdong Provincial People’s Hospital (S2024-1219-01) and was performed in accordance with the relevant guidelines.

### Subcutaneous co-injection tumour model

BMDMs were cultured in Dulbecco's modified Eagle medium (DMEM) with 10% fetal bovine serum (FBS) and macrophage colony-stimulating factor (M-CSF) (20 ng/ml) for 7 days. MMT cells were generated by treating BMDMs with A549-conditioned medium and TGFβ (5 ng/ml) for an additional 7 days. Luciferase-labelled A549 (A549-luc) cells were mixed with BMDMs or MMT cells at a 1:2 ratio (5 × 10^5^ tumour cells + 1 × 10^6^ stromal cells in 100 μl of phosphate-buffered saline (PBS)) and injected subcutaneously into the right flank of 6-week-old nude mice. Control mice received A549-luc cells alone. Tumours were harvested on day 14 for analysis.

### Cells and treatment

A549-luc and Lewis lung carcinoma cells were established through infection with UBI-MCS-EGFP-SV40-FIREFLY-Luciferase-IRES-Puromycin, and the cells were cultured with 5 μg/ml polybrene. After overnight incubation, the culture medium was replaced, and the cells were screened in 5 μg/ml puromycin. In the in vitro experiments, 250 μM 4-OI, 1 mM citraconate, 2 μg siRNA or 2 μg plasmids were added after changing the culture medium. For transfection with siRNA or plasmids, A549 cells were transfected with siRNA against human PSAT1 using INVI DNA RNA Transfection Reagent (Invigentech, CA, USA) for 24 h, as suggested by the manufacturer’s protocol.

Cells derived from bone marrow were rinsed out, filtered and cultured with M-CSF (50 ng/ml) for 24 h. Non-adherent cells were then transferred to new culture plates and cultured with M-CSF to obtain BMDMs. BMDMs were selected as a standardized primary macrophage starting population to enable controlled induction and quantitative assessment of MMT with A549-CM and TGFβ (5 ng/ml) for 5 days. The A549-CM was obtained by incubating A549 cells overnight in serum-free DMEM, followed by filtration using a 0.2-µm nylon membrane.

### NSCLC transcriptomic and survival analysis

To explore the correlation and prognostic significance of gene expression signatures across lung cancer, the GEPIA (http://gepia2.cancer-pku.cn/#correlation) and KMplot (https://kmplot.com) online platforms were employed^23,24^, both based on The Cancer Genome Atlas (TCGA) NSCLC transcriptomics and clinical data. Custom gene signatures were input into GEPIA to assess pairwise correlations between transcriptional programmes. For survival analysis, KMplot was used to evaluate the prognostic value of individual genes, with patients stratified into high-expression and low-expression groups based on the platform’s auto-selected best cutoff. Kaplan–Meier curves, hazard ratios (HRs) and log-rank *P* values were generated, and *P* <0.05 was considered statistically significant.

### Ultrahigh-performance liquid chromatograph–tandem mass spectrometry

Lipids were analysed using a Q Exactive mass spectrometer (Thermo Fisher Scientific, USA) with a CSH C18 column (1.7 μm, 2.1 × 100 mm, Waters, USA) for separation. The following gradient was used for elution: 0–2 min, 40–43% of liquid B (10 mM ammonia formate, 0.1% formic acid, 90% isopropyl alcohol and 10% acetonitrile); 2–2.1 min, 43–50% liquid B; 2.1–7 min, 50–54% liquid B; 7–7.1 min, 54–70% liquid B; 7.1–13 min, 70–99% liquid B, at a flow rate of 0.35 ml/min.

The mass spectrometric settings for positive and negative ionization modes were as follows: spray voltage, +3.8 and –3.2 kV; auxiliary gas heater temperature, 350 °C; capillary temperature, 320 °C. The full scan range was 200–2,000 m/z at a resolution of 70,000, and the automatic gain control target for mass spectrometry acquisitions was set to 3 × 10^6^ with a maximum ion injection time of 100 ms. The top three precursors were selected for subsequent mass spectrometry fragmentation with a maximum ion injection time of 50 ms and resolution of 17,500, the automatic gain control was set to 1 × 10^5^. The stepped normalized collision energy was set to 15, 30 and 45 eV. LipidSearch 4.1 SP2 software (Thermo Fisher, USA) was used for lipid identification and quantitation. Data scaling and normalization were further processed using metaX^25^.

### Conditioned medium preconditioning experiment

A549-luc cells were seeded in six-well plates at 2 × 10^5^ cells per well and cultured for 24 h. Cells were then treated for 24 h with: (1) fresh DMEM plus 10% FBS (control conditioned medium); (2) MMT-conditioned medium (MMT-CM, cells were cultured with MMT-conditioned medium, collected after 48 h, filtered through 0.22 μm); or (3) MMT-CM supplemented with 500 μM citraconate (pre-incubated for 2 h). Following treatment, cells were detached with trypsin, washed twice with PBS, and resuspended at 2 × 10^6^ cells/20 μl PBS for tibial injection. Pretreated A549-luc cells were injected into the tibial marrow cavity of mice as described in the tibial bone metastasis model. Tumour growth was monitored by bioluminescence imaging on day 14.

### Immunofluorescence staining of cultured cells

Cultured BMDMs or MMT cells were fixed with 4% paraformaldehyde for 15 min, permeabilized with 0.3% Triton X-100, and blocked with 5% bovine serum albumin. Cells were then incubated overnight at 4 °C with primary antibodies against F4/80 and αSMA, followed by fluorophore-conjugated secondary antibodies and nuclear counterstaining using DAPI. Fluorescent images were acquired using a Leica SP8 confocal microscope (Leica Microsystems) and analysed with Image J software. A comprehensive list of primary and secondary antibodies used in these experiments is provided in Supplementary Table 2.

### AAV-mediated gene silencing in vivo

The AAV HBAAV2/5-hTERT-h-siPSAT1-3xflag-ZsGreen (AAV-siPSAT1 group; 1.3 × 10^12^ viral genome/µl) was constructed and synthesized by HANBIO (Shanghai, China). AAV was administered via multiple-point injection (20 μl per tumour) on day 3 after post-A549 injection, with tumours harvested on day 14 for analysis.

### ELISA

An equal number of cells or tumour tissue of the same weight was subjected to fragmentation treatment, and the homogenates were centrifuged at 10,000 RPM for 5 min. The supernatants were recovered, aliquoted and kept at –80 °C. Itaconate content was measured using a Human itaconic acid ELISA Kit (Meimian, Yancheng, China) according to the manufacturer’s instructions.

### BrdU assay

BrdU assays were performed using a Phase-Flow™ BrdU Kit (Biolegend) following the manufacturer’s protocol. Briefly, the cells were treated with BrdU solution for 3 h before harvest, and the samples were fixed, permeabilized and treated with DNase for 1 h at 37 °C. The samples were then incubated with a BrdU antibody conjugated to phycoerythrin for 30 min. The specimens were detected by flow cytometry (Beckman Coulter). The data were analysed with FlowJo v10 (FlowJo, LLC, USA).

### Apoptosis assays

For in vitro experiments, the apoptosis of A549 cells was detected using a Vybrant FAM Caspase 3 and Caspase 7 Assay Kit (Invitrogen, CA, USA). Samples were incubated with a FAM-conjugated DEVD-FMK compound (fluorescent-labelled inhibitor of caspases) for 1 h at 37 °C. The samples were detected with a BD FACSCalibur (BD Biosciences), and the data were analysed using FlowJo v10.

### Transcriptomics analysis

Total RNA was extracted from A549 cells treated with 4-OI or without treatment using Trizol (Invitrogen). A TruSeq RNA sample preparation kit (Illumina) was used to prepare the RNA sequencing (RNA-seq) transcriptome library, which was sequenced with an Illumina HiSeq X Ten (23,150 bp read length). To identify differentially expressed genes, the expression level of each transcript was calculated using the fragments per kilobase of transcript per million mapped reads method. RNA-seq by expectation-maximization was utilized to quantify the gene abundances. EdgeR was used for differential expression analysis.

### Real-time PCR

Total RNA in cells was extracted using Trizol. RNA was reverse transcribed into cDNA by using a PrimeScriptRT Master Mix kit (TaKaRa) following the manufacturer’s protocol. mRNA levels of the target genes were normalized to mRNA levels of β-actin. Relative mRNA expression was calculated using the 2-ΔΔCq method. The primer sequences are listed in Supplementary Table 2.

### Chromatin immunoprecipitation qRT-PCR

A549 cells were treated with 4-OI (250 μM) for 4 h. Subsequently, cells were cross-linked in 37% formaldehyde and then terminated with 2.5 M glycine. Cells were then lysed, and the genome was sonicated into 200–1,000 bp fragments. The DNA cross-linking complex was immunoprecipitated with IgG antibody and ATF4 antibody, and subsequently de-cross-linked to enrich DNA, followed by quantitative real-time PCR (qRT-PCR) detection. Primer information is provided in Supplementary Table 2.

### Western blotting

Total proteins (20 μg) were separated using SDS-PAGE gels. The proteins were transferred to polyvinylidene difluoride membranes and then blocked with 5% (w/v) skim milk diluted in Tris-buffered saline with 0.1% Tween 20 for 1 h at room temperature. The membranes were then incubated with primary antibodies at 4 °C overnight. After the specimens were washed with Tris-buffered saline with 0.1% Tween 20, secondary antibodies were added and incubated at room temperature for 2 h. Densitometric analysis was performed using a ChemiDoc Touch Imaging System (Bio-Rad Laboratories). The primary and secondary antibodies used are listed in Supplementary Table 2.

### Drug affinity responsive target stability liquid chromatography–tandem mass spectrometry analysis

A549 cells were harvested on ice, lysed in ice-cold detergent-free buffer and clarified (14,000 *g*, 15 min, 4 °C). Subsequently, the protein was quantified using bicinchoninic acid and all samples were adjusted to 2 mg/ml. The lysate was split into two equal aliquots: 4-OI (250 µM) were added into one, the same volume of DMSO was added to the other. The samples were incubated for 30 min at room temperature. Freshly diluted pronase was then added at ratios of 1:100, 1:200, 1:400, 1:600, 1:800 and 1:1,000 (v/v) and allowed to be digested for 5 min at 37 °C with gentle mixing; the reaction was stopped by adding 4 × Laemmli buffer and boiling for 5 min. Equal protein amounts were loaded per lane, using 10% SDS-PAGE, and stained with SimplyBlue. Lanes were then compared: bands that remained intact in the compound lane but disappeared in the vehicle lane were indicative of protected targets. The digested peptides of each sample were desalted on C18 cartridges (Em pore SPE Cartridges C18 (standard density), bed ID 7 mm, volume 3 ml; Sigma, MO, USA), concentrated by vacuum centrifugation and reconstituted in 40 ml of 0.1% (v/v) formic acid. Phosphopeptide enrichment was conducted using a High-Select Fe-NTA Phosphopeptides Enrichment Kit according to the manufacturer’s instructions (Thermo Fisher Scientific, MA, USA). After lyophilization, the phosphopeptide peptides were resuspended in 20 ml of loading buffer (0.1% formic acid). Liquid chromatography–tandem mass spectrometry analysis was performed on a Q Exactive mass spectrometer (Thermo Scientific) coupled to an Easy nLC (Proxeon Biosystems, now Thermo Fisher Scientific) for 120 min. The mass spectrometry raw data for each sample were combined and searched using MaxQuant software for identification and quantification.

### Bioluminescence imaging

Mice were intraperitoneally injected with 75 mg/kg D-Luciferi. Bioluminescence images were acquired using the IVIS Imaging System (Perkin Elmer) at 2–5 min after injection. Analysis was conducted by measuring photon flux within the area of interest.

### Cell preparation and FACS

Primary macrophages were isolated as described. Briefly, cells derived from bone marrow were rinsed out and filtered. The flushed liquid was filtered through 100-μm and 40-μm cell strainers (BD Bioscience). Following erythrocyte elimination, the cells were resuspended in washing buffer consisting of PBS with 2% FBS. The cells were stained with antibodies for 30 min at 4 °C in the dark and then isolated by fluorescence-activated cell sorting (FACS) using a FACSAria III system (BD Biosciences). The data were analysed with FlowJo v10. Information on the antibodies is listed in Supplementary Table 2.

### FACS isolation of bone metastasis cell subsets

Primary macrophages were isolated as described. Tibiae with established A549 fluorescein isothiocyanate (FITC)-tagged bone metastases were harvested, flushed with PBS, and digested with collagenase IV (2 mg/ml) and Dispase II (1 mg/ml) at 37 °C for 45 min. Single-cell suspensions were obtained by passing through a 70 μm strainer, followed by red blood cell lysis. A549 tumour cells were identified by FITC fluorescence. Haematopoietic cells were gated as CD45^+^, with MMT cells defined as CD45^+^CD68^+^αSMA^+^, macrophages as CD45^+^CD11b^+^F4/80^+^, neutrophils as CD45^+^CD11b^+^Ly6G^+^Siglec-F⁻, T cells as CD45^+^CD3^+^CD11b⁻CD19⁻, and B cells as CD45^+^CD3⁻CD11b⁻CD19^+^. Following erythrocyte elimination, cells were resuspended in washing buffer consisting of PBS with 2% FBS. The cells were stained with antibodies for 30 min at 4 °C in the dark, and were then isolated by a FACSAria III system (BD Biosciences). The data were analysed with FlowJo v10. Sorted cells were lysed for ELISA-based itaconate quantification. Information on the antibodies is listed in Supplementary Table 2.

### Histochemistry and cytochemistry

Briefly, freshly frozen tumour tissues were sectioned into 8-μm sections. The sections were fixed with 4% PFA for 5 min followed by permeabilization in 0.5% Triton X-100 (Solarbio) in PBS for 5 min. The sections were blocked for 1 h at 37 °C in PBS containing 10% normal donkey serum, 3% bovine serum albumin, and 0.1% Triton X-100 and incubated with primary antibodies at 4 °C overnight. Excessive primary antibodies were then washed away and the sections were cultured in secondary antibodies conjugated to AF488, Cy3, or phalloidin conjugated to AF647 or TRITC. The sections were then counterstained with Hoechst 33342 or DAPI (Beyotime). The primary and secondary antibodies used are listed in Supplementary Table 2.

### Statistical analysis

The results are shown as the mean ± SDs. The Student *t*-test was used to compare two groups. For more than two groups, one-way analysis of variance (ANOVA) was used. Statistical significance was considered at *P* <0.05. All experiments were repeated at least three times.

## Results

### MMT promotes tumour progression and is positively correlated with clinical lung cancer bone metastasis

**Fig. 1:**
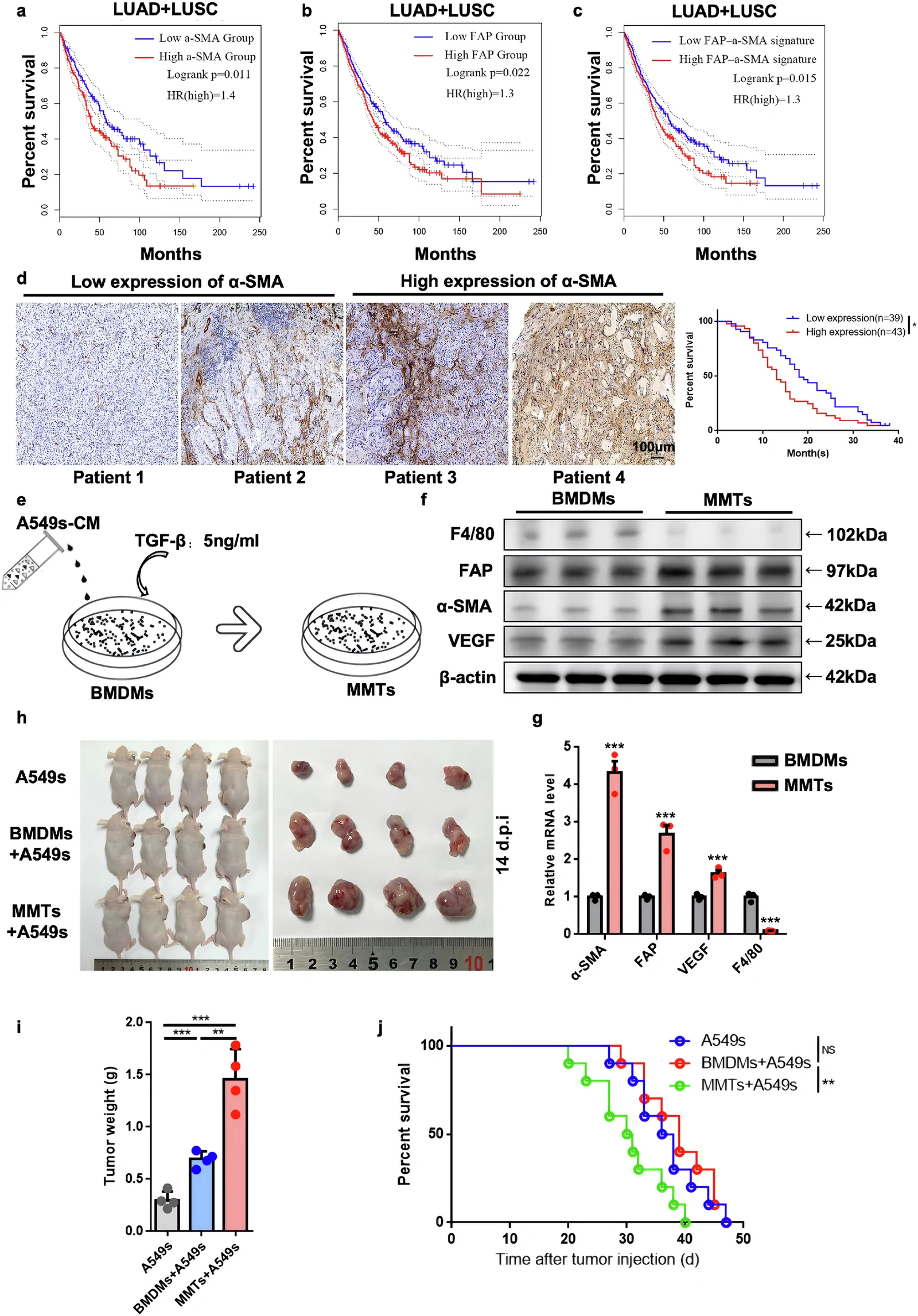
MMT promotes tumour progression and positively correlated with clinical lung cancer bone metastasis. Kaplan–Meier analysis of survival in patients with lung cancer was performed based on the expression of αSMA (ACTA2; part **a**), fibroblast-activating protein (FAP; part **b**) and their combined signature (part **c**). Representative immunohistochemistry images of αSMA (part **d**, left panel; scale bar, 100 μm) and Kaplan–Meier survival analysis of αSMA (part **d**, right panel). Schematic diagram of macrophage-to-myofibroblast transformation (MMT) induction in vitro (part **e**). Western blotting analysis (part **f**) and quantitative real-time PCR (part **g**) of F4/80, FAP, αSMA and VEGF expression in bone marrow-derived macrophages (BMDMs) and MMT cells. Representative images of subcutaneous tumours in mice 14 days after injection of A549 cells alone or in combination with BMDMs or MMT cells (part **h**). Ex vivo tumour weights (part **i**). Kaplan–Meier survival curve of mice (part **j**).

To systematically evaluate the clinical significance of MMT in lung cancer, the GEPIA and KMplot online platforms, both of which are based on TCGA NSCLC transcriptomics and clinical data, were first employed^23,24^. Univariate Cox analysis revealed that patients with high expression of αSMA (ACTA2; the specific biomarker of myofibroblasts) had lower overall survival rates (HR 1.4; *P* = 0.011) (Fig. 1a). The expression of fibroblast-activating protein (FAP) was also negatively correlated with survival (HR 1.3; *P* = 0.022) (Fig. 1b). In terms of the median expression of ACTA2 and FAP, the median survival time in the dual-high-expression group was significantly lower than that in the dual-low-expression group (*P* = 0.015) (Fig. 1c). In lung cancer specimens collected from patients with lung cancer with bone metastases who underwent resection at Guangdong Provincial People's Hospital between 2019 and 2025, the median survival time of patients with strongly positive αSMA expression (H score ≥180; *n* = 43) confirmed by immunohistochemistry was much lower than that of patients with low αSMA expression (H score <90; *n* = 39) (Fig. 1d), consistent with the trend observed in the public database. These clinical findings suggest that elevated αSMA expression — a hallmark of activated CAFs — is closely associated with a more aggressive tumour phenotype and poorer prognosis in lung cancer with bone metastasis. Notably, accumulating evidence indicates that CAFs can originate from multiple cellular sources within the tumour microenvironment, among which MMT has emerged as an important contributor to the fibroblast pool, particularly under profibrotic and tumour-promoting conditions^26,27^. Since the role of TGFβ in CAF transformation has been confirmed^28,29^, TGFβ was used herein to induce MMT cells in vitro. BMDMs from C57BL/6 mice were treated with conditioned medium from A549 cells (A549-CM) supplemented with 5 ng/ml TGFβ for 7 days (Fig. 1e). Western blot analysis revealed that the expression of F4/80 was downregulated after induction, while the expression of FAP, αSMA and VEGF was significantly upregulated (Fig. 1f). Furthermore, qRT-PCR verified these changes (Fig. 1g). To determine the optimal ratio of tumour cells to macrophage lineage cells for in vivo co-injection, preliminary experiments comparing different A549-to-BMDM ratios (1:1, 1:2 and 1:4) were performed. The 1:2 ratio yielded the most suitable experimental conditions (Supplementary Fig. 1a), characterized by robust and reproducible tumour formation and a pronounced macrophage-mediated tumour-promoting effect within an ethically manageable tumour burden. In contrast, the 1:1 ratio resulted in relatively modest stromal effects, whereas increasing the ratio to 1:4 did not further enhance tumour growth but instead introduced greater variability among individual mice. Based on these optimization results, a ratio of 1:2 (tumour cells-to-macrophage lineage cells) was selected for subsequent experiments.

Immunofluorescence staining revealed that the mean fluorescence intensity of αSMA increased, whereas that of F4/80 decreased (Supplementary Fig. 1b). These results confirmed the stable MMT phenotype. qRT-PCR revealed that the expression of *CDC20*, *CDK1*, *CDC7*, *CCND1* and other proliferation genes was greater in the MMT group than in the BMDM group^30–32^. Moreover, the expression of the tumour suppressor genes *CDKN1A*, *CDKN2A* and *p53* decreased (Supplementary Fig. 1c), suggesting a pro-tumorigenic transcription^33–35^. In vivo, A549 cells alone or A549 cells together with BMDMs or MMT cells mixed at a ratio of 1:2 were injected into the right armpit of nude mice. After 14 days, the average tumour volume in the MMT group was 1,087 ± 124 mm^3^, which was significantly greater than that in A549 cell alone (394 ± 63 mm^3^) or A549 cells with BMDMs (521 ± 71 mm^3^) (Fig. 1h). In vitro weighing revealed that the average tumour weight in the MMT group was significantly greater than that in A549 cells alone or in A549 cells with BMDMs (Fig. 1i). Moreover, survival analysis revealed that the median survival time of mice in the MMT group was only 30 days, whereas that of mice in the A549 and A549 plus BMDMs groups were 38 days and 40 days, respectively (Fig. 1j). Therefore, by acquiring a myofibroblast phenotype and secreting VEGF, as well as other pro-angiogenic factors such as bFGF and PDGF, MMT cells can considerably increase the growth and tumour load of lung cancer cells both in vivo and in vitro.

### Metabolic reprogramming of MMT through high *IRG1* and itaconate expression drives lung cancer osteolytic progression

**Fig. 2:**
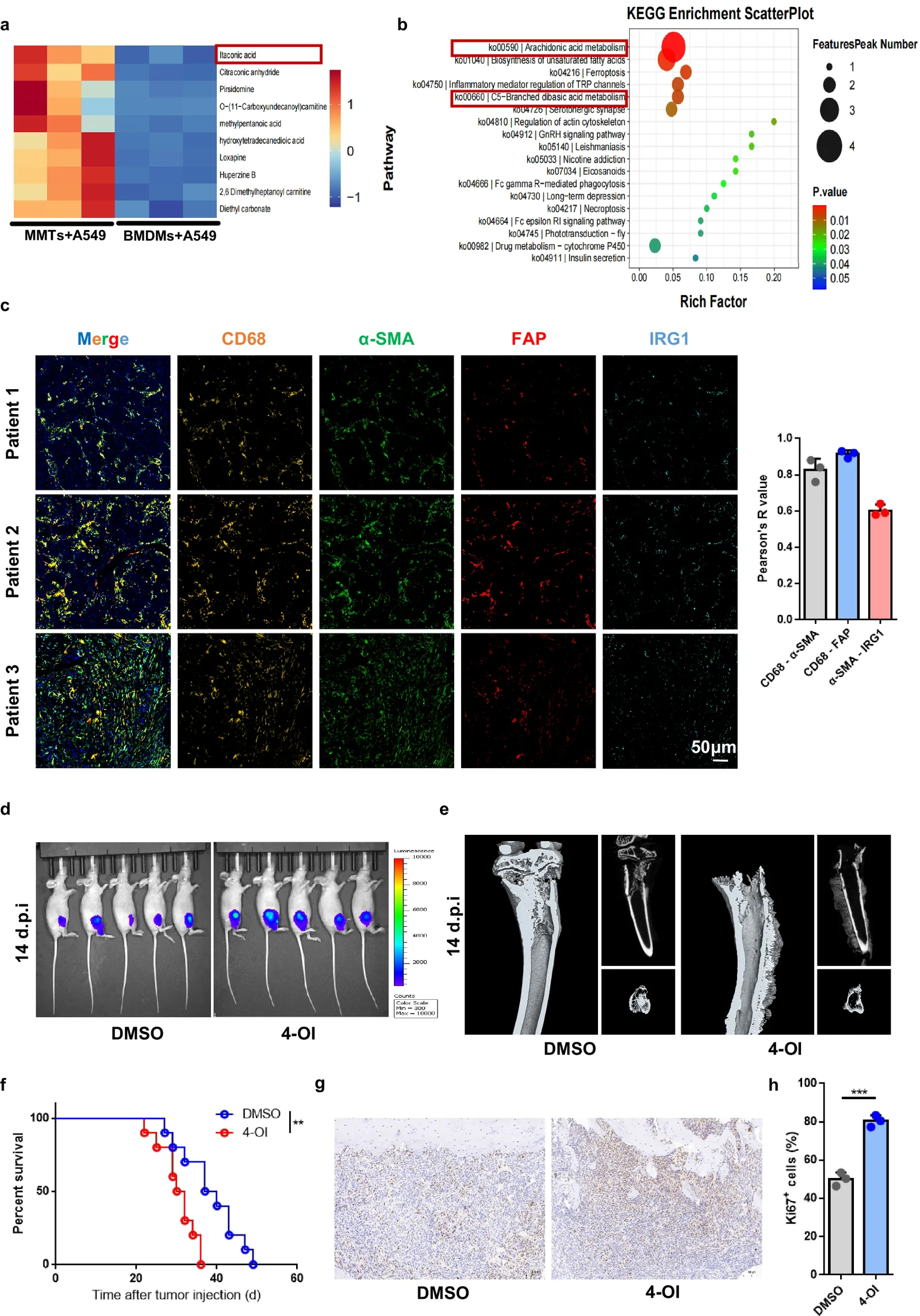
MMT-derived cancer-associated fibroblasts are metabolically reprogrammed to acquire an *IRG1*–itaconate-high phenotype that promotes lung cancer osteolytic progression. Untargeted metabolomics of tumour tissues from subcutaneous tumours formed by co-injection of Luciferase-labelled A549 (A549-luc) cells with bone marrow-derived macrophages (BMDMs) or macrophage-to-myofibroblast transformation (MMT) cells (1:2 ratio), harvested on day 14 (part **a**). KEGG enrichment (part **b**). Representative multiplex immunofluorescence images and co-localization analysis of bone metastasis specimens from patients with lung cancer showing co-staining of CD68, αSMA, fibroblast-activating protein (FAP) and immune-responsive gene 1 (IRG1). Scale bars, 50 μm (part **c**). Bioluminescence imaging of A549-luc bone metastatic mice treated with dimethyl sulfoxide (DMSO) or 4-octyl itaconate (4-OI) (part **d**). Representative micro-CT images (part **e**). Survival times of A549-luc tumour-bearing mice treated with or without 4-OI (part **f**). Representative images and quantification of Ki67 staining. Scale bar, 100 μm (parts **g**, **h**).

Given that tumour-associated myeloid cells, such as MMT cells and BMDMs, can profoundly reshape the metabolic landscape of the tumour microenvironment, the metabolic alterations they induce were characterized herein. To uncover the metabolic reprogramming underlying MMT-induced or BMDM-induced tumour progression and identify potential therapeutic targets, metabolomic profiling of tumour tissues treated with MMT cells or BMDMs was performed. Heatmaps revealed a marked accumulation of itaconate in MMT cells compared with that in BMDMs (Fig. 2a). KEGG pathway analysis revealed significant enrichment in arachidonic acid metabolism and C5-branched dibasic acid metabolism — two pathways closely intertwined with itaconate biology (Fig. 2b). Notably, itaconate, derived from the TCA cycle intermediate *cis*-aconitate, can modulate inflammatory responses by inhibiting succinate dehydrogenase and influencing eicosanoid production, thereby intersecting with both arachidonic acid-derived lipid mediator synthesis and the metabolism of C5-branched dicarboxylic acids^21^. To validate the clinical relevance of MMT in human disease, multiplex immunofluorescence staining was performed on bone metastasis specimens from patients with lung cancer. Co-staining was conducted using the macrophage marker CD68, myofibroblast markers αSMA and FAP, and IRG1, a key metabolic enzyme responsible for itaconate production. Representative images revealed a distinct population of cells co-expressing CD68 with αSMA and/or FAP within the metastatic bone microenvironment (Fig. 2c). These positive cells were predominantly localized in stromal regions surrounding tumour nests, suggesting their involvement in tumour–stroma interactions. Quantitative co-localization analysis demonstrated a significant proportion of CD68^+^ macrophages co-expressing αSMA and FAP, supporting the presence of a macrophage population exhibiting myofibroblast-like features. Notably, *IRG1* expression was markedly enriched in these CD68^+^αSMA^+^ and/or FAP^+^ cells, indicating activation of the itaconate-producing metabolic pathway within this transitional cell population. Given that IRG1 catalyses the production of itaconate, a metabolite implicated in immunometabolic reprogramming, its co-expression with myofibroblast markers suggests that metabolic remodelling is closely associated with the acquisition of myofibroblast-like phenotypes. To further investigate the metabolic characteristics of distinct cellular populations within the tumour microenvironment, intracellular itaconate levels were quantified across different cell subsets. Single-cell suspensions were prepared from dissociated tumour tissues and subjected to FACS based on established lineage-specific markers to isolate macrophages, MMT cells, neutrophils, tumour cells, T cells and B cells (Supplementary Fig. 2a,b). Equal numbers of each sorted cell population were subsequently lysed, and intracellular itaconate levels were measured by ELISA. The highest content of itaconate was detected in the MMT cell subset, followed by macrophages and neutrophils, whereas A549 cells, T cells and B cells contained negligible amounts (Supplementary Fig. 2c). Concordantly, itaconate content in the tumour and peripheral blood in the MMT cell group was greater than that in the tumour and blood of mice in the BMDM group (Supplementary Fig. 2d). To test the function of itaconate, A549 cells and tumour-bearing mice were treated with the cell-permeable itaconate derivative 4-OI, which is released intracellularly in the form of itaconate^17^. qRT-PCR revealed significant upregulation of key cell-cycle-promoting and anti-apoptotic genes, including *CDC20*, *CDK1*, *CDCA3*, *CENPA*, *CDC7*, *CCND1* and *BCL2*. Concurrently, the critical tumour suppressors *TP53* (*p53*), *CDKN1A* (*p21*) and *CDKN2A* (*p16*) were reciprocally downregulated, collectively indicating enhanced proliferative capacity, dysregulated cell-cycle progression and evasion of growth suppression (Supplementary Fig. 3a). Furthermore, flow cytometry of BrdU revealed increased proliferation (Supplementary Fig. 3b) and decreased Caspase 3 activity (Supplementary Fig. 3c). A549-luc cells were injected in situ into the tibia to generate mice with lung cancer bone metastases. The bioluminescence of the tumour site was significantly enhanced after 4-OI treatment (Fig. 2d and Supplementary Fig. 4a). The tibia was removed for micro-CT detection and three-dimensional reconstruction. 4-OI induced increased bone destruction and deterioration of bone microstructure, revealing obvious osteolytic defects at the proximal end, interruption in cortical continuity and multiple perforations in the bone marrow cavity (Fig. 2e). Specifically, bone mineral density was significantly reduced and bone volume per total volume was markedly decreased, indicating impaired mineralization and substantial loss of bone mass. Concurrently, trabecular number declined, trabecular thickness was reduced and trabecular separation increased, collectively suggesting a sparse and atrophic trabecular architecture with widened inter-trabecular spaces and compromised connectivity of the bone (Supplementary Fig. 4b). In addition, in the 4-OI group, shorter survival times (Fig. 2f) and higher positive rates of Ki67 staining (Fig. 2g, h) were recorded.

### IRG1 promotes tumour progression and bone metastasis through MMT-dependent metabolic reprogramming

**Fig. 3:**
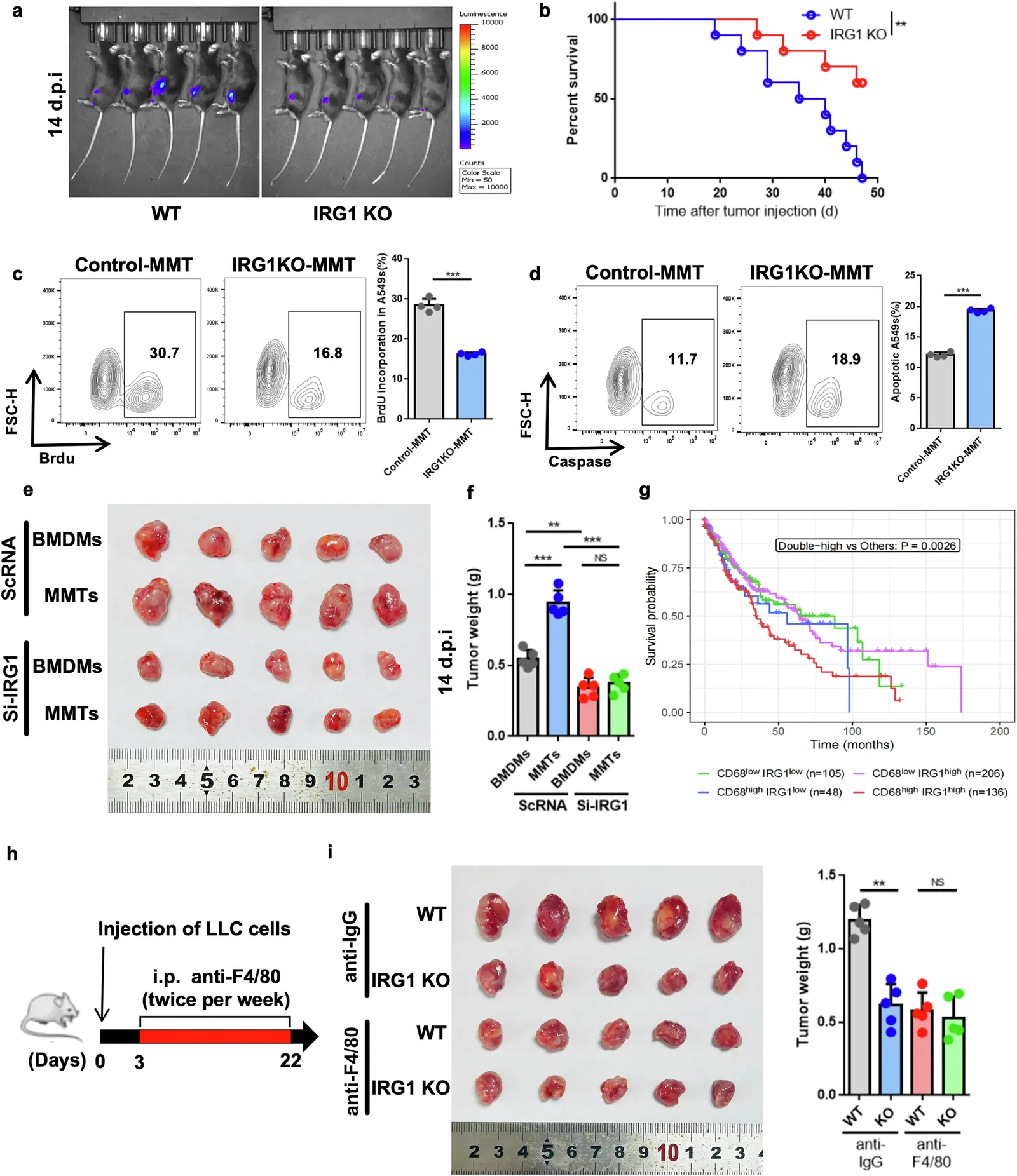
IRG1 promotes tumour progression and bone metastasis through MMT-dependent metabolic reprogramming. **a** Representative bioluminescence images of tumour-bearing mice in *Irg1* wild-type (WT) and *Irg1*-knockout (KO) groups. **b** Kaplan–Meier survival curves. **c** Flow cytometric analysis of tumour cell proliferation in Control macrophage-to-myofibroblast transition (MMT) and *Irg1*-KO MMT (*Irg1*-KO-MMT) groups, assessed by BrdU incorporation. **d** Flow cytometric analysis of apoptosis in Control-MMT and *Irg1-*KO-MMT groups, assessed by caspase-positive staining. **e** Representative gross images of excised tumours from each group at the experimental end point. Luciferase-labelled (A549-luc) cells were co-injected with bone marrow-derived macrophages (BMDMs) or MMT cells transfected with scrambled RNA (scRNA) or *si-Irg1*. **f** Quantitative analysis of tumour weight inhibition following *Irg1-*KO, showing a significantly greater effect in MMT cells compared with BMDMs. **g** Kaplan–Meier survival analysis of patients with lung cancer in The Cancer Genome Atlas dataset stratified by CD68 and *IRG1* expression levels, indicating poorer prognosis in the *CD68*^high^*IRG1*^high^ group. **h** Schematic illustration of macrophage neutralization using anti-F4/80 antibody in vivo. **i** In vivo bioluminescence analysis of bone metastasis in WT and *Irg1*-KO mice with or without macrophage depletion. Macrophage neutralization significantly reduced tumour burden and abolished the difference between WT and *Irg1*-KO groups.

Since IRG1 is the rate-limiting enzyme for itaconate synthesis^21^, *Irg1* wild-type and *Irg1*-knockout C57BL/6 mice were used to confirm its role. Bioluminescence of tumours was attenuated (Fig. 3a and Supplementary Fig. 4c), and median survival was prolonged (Fig. 3b) in the *Irg1-*knockout mice. To determine whether IRG1 is required for itaconate production in MMT cells, intracellular itaconate levels were measured in wild-type MMT and *IRG1*-knockout MMT groups. IRG1 deficiency led to a marked reduction in itaconate levels, indicating that IRG1 is essential for maintaining itaconate production in MMT cells (Supplementary Fig. 4d). To further assess the impact of IRG1 on tumour cell behaviour within the MMT context, flow cytometric analyses comparing Control-MMT and *IRG1*-knockout MMT groups were performed. BrdU incorporation assays revealed a significant reduction in proliferative activity in the *IRG1-*knockout MMT group relative to controls (Fig. 3c). Consistently, apoptosis analysis demonstrated a marked increase in caspase-positive cells upon IRG1 depletion (Fig. 3d), suggesting that loss of IRG1 impairs MMT-mediated tumour-supportive functions by suppressing proliferation and promoting apoptosis. To further confirm the efficiency of *IRG1* knockdown at the transcriptional level and validate the specificity of siRNA-mediated silencing, *IRG1* mRNA expression was assessed. qRT-PCR analysis showing *IRG1* mRNA levels in BMDMs and MMT cells transfected with control or siRNA targeting *IRG1* (si-IRG1). *IRG1* expression was significantly reduced in both cell types upon si-IRG1 treatment, confirming effective knockdown (Supplementary Fig. 4e). To directly determine whether MMT-derived, rather than conventional macrophage-derived, IRG1–itaconate drives tumour progression, a 2 × 2 factorial design experiment combining cell identity (BMDMs versus MMT cells) with *IRG1* knockdown was performed. Specifically, A549-luc cells were co-injected with either BMDMs or MMT cells transfected with control siRNA or si-IRG1. Tumour growth was then monitored longitudinally in vivo (Fig. 3e). In the control groups, co-injection with MMT cells resulted in markedly accelerated tumour growth compared with BMDMs, consistent with their enhanced tumour-promoting capacity. Notably, IRG1 silencing led to a substantial suppression of tumour growth in the MMT group, whereas only a modest reduction was observed in the BMDM group. Quantitative analysis revealed that the inhibitory effect of *IRG1* knockdown was significantly greater in MMT cells than in BMDMs (Fig. 3f), indicating a heightened dependency of MMT cells on IRG1-mediated metabolic reprogramming. Hence, while IRG1 contributes to the tumour-promoting activity of both macrophages and MMT cells, its functional impact is substantially amplified following MMT transition, supporting a predominant role of MMT-derived IRG1–itaconate in driving tumour progression.

Notably, RNA-seq data of patients with lung cancer from TCGA database indicated that high expression of *IRG1* in macrophages (*CD68*^high^*IRG1*^high^) predicted poor prognoses compared with low expression (*CD68*^high^*IRG1*^low^, *CD68*^low^*IRG1*^high^ and *CD68*^low^*IRG1*^low^) (Fig. 3g). These results prompted the exploration of whether macrophage-derived IRG1 is involved in the progression of bone metastases in lung cancer. To test this hypothesis, macrophages were neutralized with anti-F4/80 antibodies (Fig. 3h). With the gating strategy shown in Supplementary Fig. 4f, flow cytometry confirmed that more than 70% of macrophages were neutralized (Supplementary Fig. 4g). In vivo results revealed that lung cancer bone metastases in macrophage-deficient wild-type mice were significantly inhibited, while there was almost no difference between wild-type and *Irg1*-knockout mice after macrophage neutralization (Fig. 3i). Hence, IRG1 acts as a critical immunometabolic enzyme and may contribute to the progression of lung cancer bone metastasis through metabolic reprogramming. This functional role further suggests that targeting IRG1-driven itaconate metabolism may represent a potential therapeutic strategy. Citraconate, a natural competitive inhibitor of IRG1, is used to block itaconate synthesis^36^. To further evaluate the functional contribution of the IRG1–itaconate axis in vivo, itaconate production was pharmacologically inhibited using citraconate in MMT-CM mice. Liquid chromatography–mass spectrometry analysis confirmed the effectiveness of this intervention, demonstrating an approximately 75% reduction in intratumoural itaconate levels (Supplementary Fig. 5a). Functionally, this metabolic inhibition translated into a marked suppression of tumour progression. Citraconate treatment significantly reduced tumour burden, as evidenced by decreased bioluminescence signals (Supplementary Fig. 5b,c), and notably prolonged overall survival, extending median survival from 32 to 46 days (Supplementary Fig. 5d). Consistent with these phenotypic changes, molecular analysis further revealed that citraconate treatment reversed the upregulation of proliferation-associated genes induced by MMT (Supplementary Fig. 5e), supporting a critical role of itaconate-driven metabolic reprogramming in mediating MMT-associated tumour-promoting effects.

### Itaconate promotes cancer proliferation via the upregulation of *PSAT1* expression

**Fig. 4:**
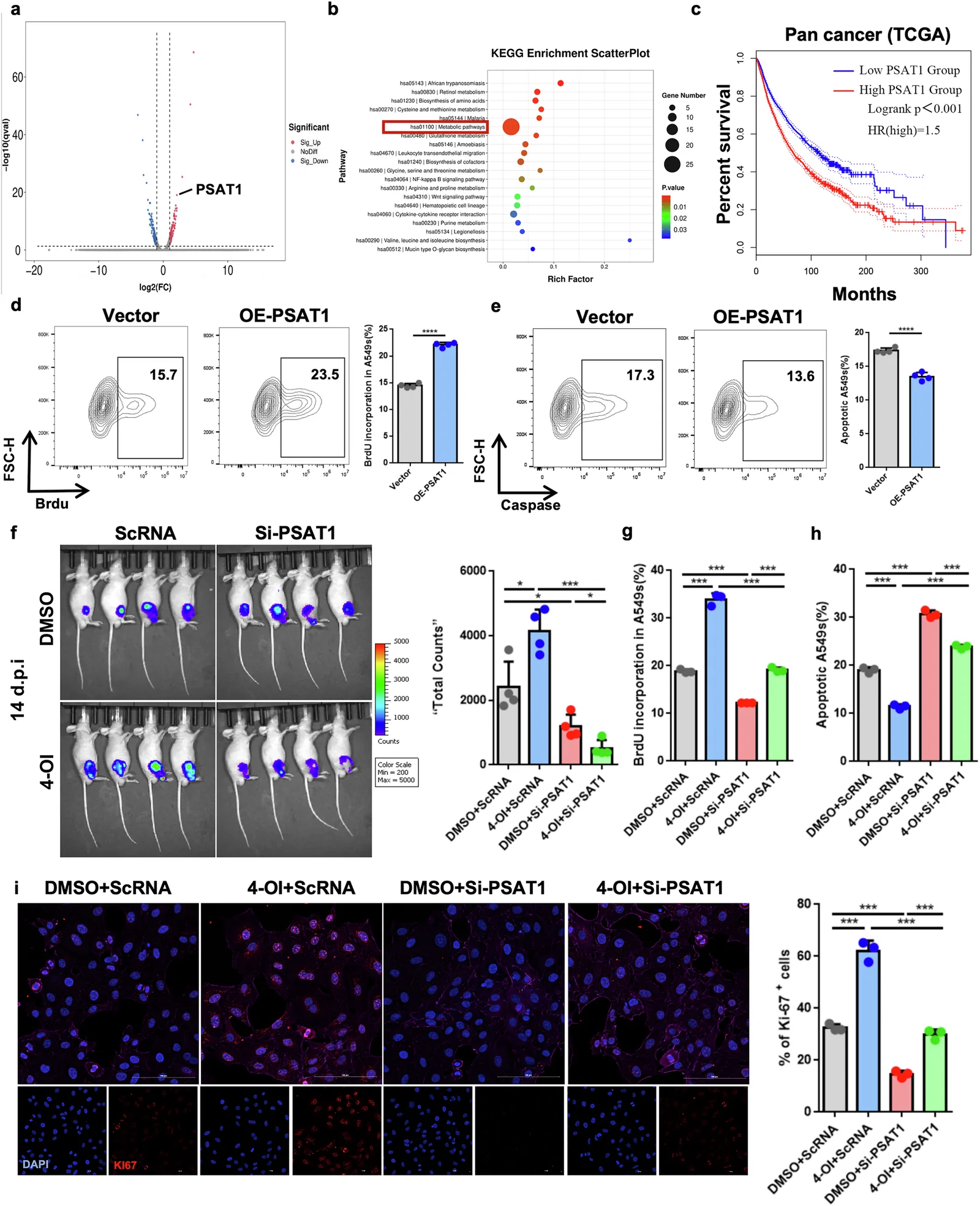
PSAT1 is a critical metabolic node that links itaconate to tumour aggressiveness. **a** Volcano plot of RNA sequencing. **b** KEGG enrichment. **c** Kaplan–Meier survival curve for *PSAT1* expression across cancers in The Cancer Genome Atlas (TCGA) cohort. **d** Representative flow cytometry images of BrdU staining. **e** Representative flow cytometry images of caspase expression. **f** In vivo bioluminescence images. **g** Quantification of BrdU flow cytometry. **h** Quantification of caspase flow cytometry. **i** Representative images and mean fluorescence intensity of Ki67 staining. Scale bar, 100 μm. PSAT1, phosphoserine aminotransferase 1.

To identify the core molecular target mediating the biological effects of itaconate, RNA-seq was performed on A549 cells stimulated with 4-OI for 24 h. Differential expression analysis revealed hundreds of significantly altered transcripts; however, *PSAT1* emerged as one of the most robustly upregulated genes (Fig. 4a and Supplementary Fig. 6a). Given its pivotal role in serine biosynthesis — a pathway increasingly recognized for supporting redox balance, nucleotide synthesis and tumour cell proliferation — *PSAT1* was prioritized for further investigation. Consistently, KEGG pathway enrichment highlighted metabolic pathways, particularly those linked to amino acid and one-carbon metabolism (Fig. 4b and Supplementary Fig. 6b), reinforcing the potential relevance of *PSAT1* in itaconate-driven tumour promotion. To test this hypothesis, expression of *PSAT1* and prognoses in a pancancer dataset from TCGA were analysed. The results confirmed that high *PSAT1* expressions correlated with reduced overall survival (HR 1.5; *P* <0.001) (Fig. 4c), and qRT-PCR confirmed the 4-OI-induced increase in *PSAT1* mRNA expression (Supplementary Fig. 6c). To verify the relationship between *PSAT1* and itaconate-driven proliferation, a cell model was constructed in which *PSAT1* was knocked down (Supplementary Fig. 6d) or overexpressed (Supplementary Fig. 6e). Flow cytometry revealed that *PSAT1* overexpression increased the proliferation index (Fig. 4d) and decreased the activity of caspase 3 (Fig. 4e), whereas *PSAT1* knockout completely abolished itaconate-induced tumour growth (Fig. 4g and Supplementary Fig. 6f) and apoptosis resistance (Fig. 4h and Supplementary Fig. 6g). qRT-PCR revealed similar results. *PSAT1* knockout decreased the expression of oncogenes and increased the expression of tumour suppressor genes (Supplementary Fig. 6h). In vivo, the knockdown of *PSAT1* reversed the tumour proliferation induced by itaconate, resulting in a marked reduction in the bioluminescence signal (Fig. 4f). Moreover, tumour cells treated with 4-OI exhibited substantial positive expression of Ki67, whereas *PAST1* silencing greatly reduced its expression (Fig. 4i). Collectively, these results establish *PSAT1* as the essential downstream effector through which itaconate accelerates tumour proliferation.

### HSPA8 is the direct target through which itaconate upregulates *PSAT1*

**Fig. 5:**
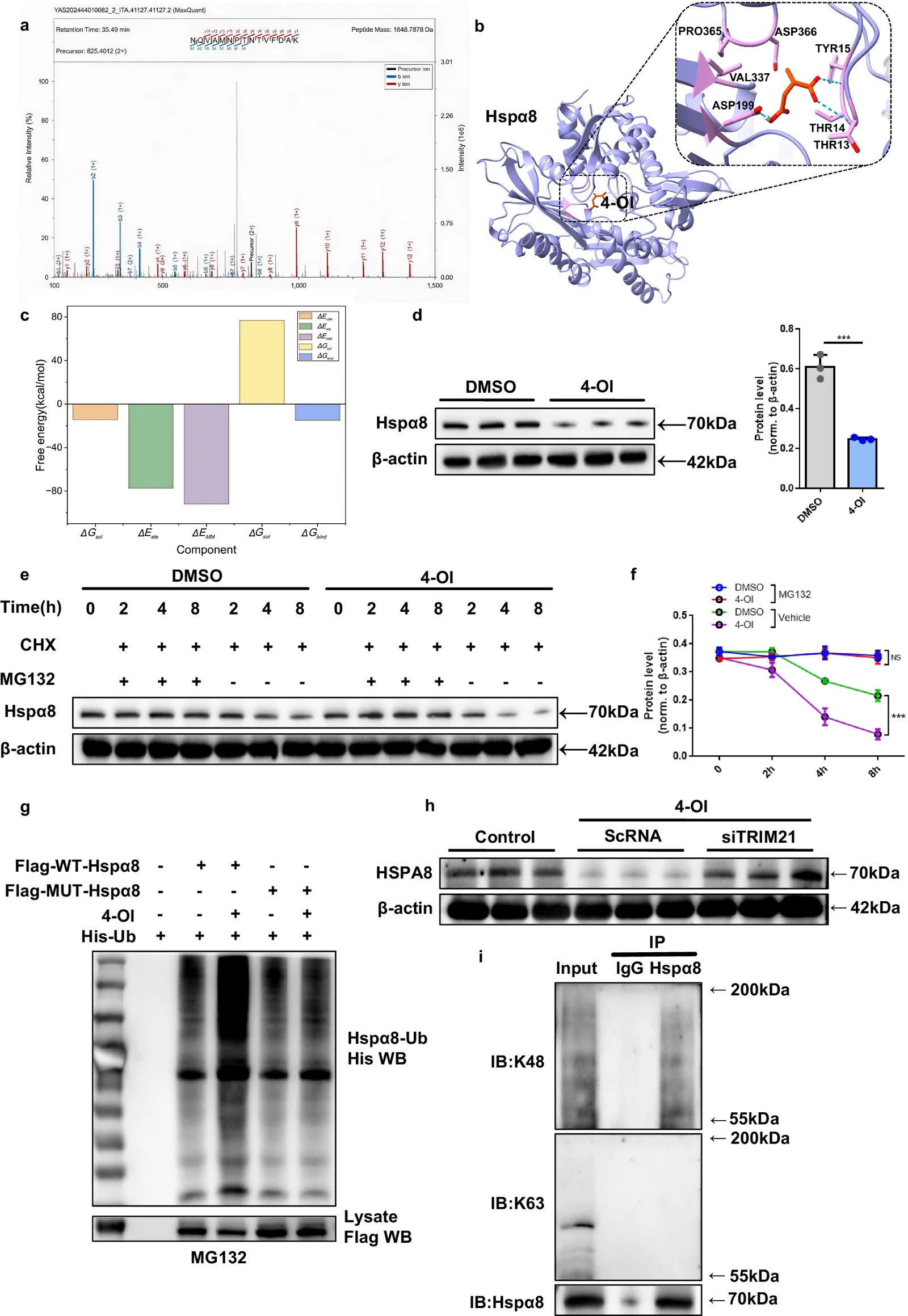
Itaconate triggers ubiquitin-dependent degradation of HSPA8. **a** Liquid chromatography–tandem mass spectrometry identification revealed that the protected band contained heat shock protein family A member 8 (HSPA8) as the most prominent band without competitive pollutants. **b** Molecular docking diagram of itaconate binding with HSPA8. **c** Molecular dynamics free energy decomposition of itaconate–HSPA8 binding. **d** Western blotting analysis of the expression of HSPA8 in A549 cells treated with 4-octyl itaconate (4-OI). **e** Western blotting analysis of the protein stability of HSPA8 in A549 cells treated with 4-OI or dimethyl sulfoxide (DMSO). **f** Quantification of the expression of HSPA8 in part **e**. **g** Immunoblotting revealed that 4-OI significantly reduced the ubiquitination band of HSPA8 in His-ubiquitin pull-down product in A549 cells with flag-WT-*HSPA8*, whereas A549 cells with flag-MUT-*HSPA8* were not sensitive to 4-OI. MG132 was used as a positive control to restore the ubiquitination signal, and β-actin was used as an internal reference. **h** Immunoblot analysis of HSPA8 protein levels in A549 cells transfected with control small interfering RNA (siRNA; si-Control) or siRNA targeting TRIM21 (si-TRIM21), followed by treatment with 4-OI or DMSO for 24 h. TRIM21 knockdown markedly attenuated the 4-OI-induced reduction of HSPA8 protein levels.**i** Ubiquitin linkage-specific analysis of HSPA8. Cell lysates from A549 cells treated with 4-OI or DMSO were subjected to co-immunoprecipitation using anti-HSPA8 antibody, followed by immunoblotting with K48-linkage-specific and K63-linkage-specific ubiquitin antibodies. 4-OI treatment markedly increased K48-linked ubiquitination of HSPA8, whereas K63-linked ubiquitination showed minimal changes.

Given that itaconate is a cell-permeable immunometabolite lacking intrinsic DNA-binding capacity, it is unlikely to directly induce *PSAT1* mRNA expression. Instead, itaconate likely exerts its effects by modulating the activity or stability of specific protein targets that, in turn, regulate *PSAT1* transcription or mRNA stability. To delineate this upstream mechanism, drug affinity responsive target stability coupled with ultrahigh-performance liquid chromatography–tandem mass spectrometry were employed to globally identify cellular proteins that physically interact with 4-OI, thereby uncovering potential mediators of itaconate-driven *PSAT1* upregulation. Results showed that the protected band contained HSPA8, which was the most prominent band without competitive pollutants (Fig. 5a), positioning the chaperone as a candidate direct target. Molecular docking revealed that 4-OI inserts into the ATPase pocket of HSPA8, forming a hydrogen-bond network with ASP199, THR13, THR14 and TYR15 (Fig. 5b), which is predicted to block nucleotide exchange and destabilize the protein. Molecular dynamics free energy decomposition revealed that itaconate–HSPA8 binding was driven mainly by strong electrostatic adn van der Waals forces, and the total free energy (ΔG_bind_) was approximately –40 kcal/mol, indicating that the complex of itaconate and HSPA8 was highly stable (Fig. 5c). Western blotting further confirmed that, compared with the control treatment, 4-OI treatment reduced the expression of HSPA8 by nearly 50% (Fig. 5d). Next, a protein stability test was performed. A549 cells were treated with DMSO or 4-OI with or without MG132 after cycloheximide-blocked synthesis of new proteins. Western blot analysis revealed that 4-OI significantly accelerated the degradation of HSPA8 (half-life ~2 h), whereas MG132 completely inhibited this effect, confirming that 4-OI promoted the degradation of HSPA8 through the proteasome pathway (Fig. 5e, f). To determine whether itaconate directly promotes the ubiquitination and subsequent proteasomal degradation of HSPA8, A549-luc cells were co-transfected with plasmids encoding His-tagged ubiquitin (His-Ub) and either FLAG-tagged wild-type *HSPA8* or a binding-deficient mutant *HSPA8*engineered to disrupt the predicted 4-OI interaction site. Following treatment with 4-OI, a His-tag pull-down assay coupled with western blotting revealed a significant increase in polyubiquitinated wild-type *HSPA8*, which was further enhanced upon co-treatment with the proteasome inhibitor MG132, consistent with proteasome-dependent degradation (Fig. 5g).

In contrast, ubiquitination of the *HSPA8* mutant was markedly attenuated, suggesting that 4-OI-induced HSPA8 ubiquitination depends on its direct interaction with HSPA8 at the mutated binding site. Given this interaction-dependent ubiquitination, the upstream E3 ubiquitin ligase responsible for mediating this process was identified and the nature of the ubiquitin modification was further characterized, as E3 ligases are key determinants of substrate specificity and ubiquitin linkage type in protein degradation pathways^37^. To identify the E3 ubiquitin ligase responsible for HSPA8 ubiquitination upon itaconate treatment, co-immunoprecipitation (Co-IP) assays were performed in A549 cells treated with 4-OI or DMSO for 24 h. HSPA8 was immunoprecipitated, and its interaction with candidate E3 ligases was examined by immunoblotting. Among the tested candidates, TRIM21 exhibited a markedly enhanced association with HSPA8 upon 4-OI treatment, whereas CHIP and Parkin showed relatively weaker or negligible interactions (Supplementary Fig. 7a). To further determine the functional relevance of TRIM21 in mediating HSPA8 degradation, siRNA-mediated knockdown experiments were performed. Silencing of TRIM21 significantly attenuated the 4-OI-induced reduction of HSPA8 protein levels, indicating that TRIM21 is required for HSPA8 degradation under itaconate stimulation (Fig. 5h and Supplementary Fig. 7b,c). The ubiquitin linkage type involved in this process was subsequently investigated. Co-IP of HSPA8 followed by immunoblotting with linkage-specific ubiquitin antibodies revealed a substantial increase in K48-linked ubiquitination upon 4-OI treatment, whereas K63-linked ubiquitination showed minimal changes (Fig. 5i). These findings suggest that 4-OI promotes proteasomal degradation of HSPA8 via TRIM21-mediated K48-linked ubiquitination. Hence, TRIM21 is the primary E3 ligase responsible for HSPA8 ubiquitination in response to itaconate, establishing a mechanistic link between itaconate signalling and proteasome-dependent degradation of HSPA8.

Notably, the HSPA8 mutant, which harbours a mutation specifically disrupting the 4-OI binding site while leaving its intrinsic chaperone function intact, did not respond to 4-OI (Supplementary Fig. 7d,e), confirming that itaconate accelerates HSPA8 degradation by promoting its ubiquitination in a binding-dependent manner. Furthermore, overexpression of wild-type *HSPA8* effectively reversed the 4-OI-induced increase in cell proliferation and resistance to apoptosis (Supplementary Fig. 7f,i) and restored normal transcriptional programmes and apoptotic responses. In comparison, the binding-deficient HSPA8 mutant, despite being functionally competent, completely lost this protective ability (Supplementary Fig. 7j). Collectively, these findings demonstrate that HSPA8 serves as a critical molecular node through which itaconate exerts its tumour-promoting effects, not by globally disabling HSPA8 function but by specifically targeting it for ubiquitin-mediated degradation upon direct interaction. This positions HSPA8 not merely as a passive substrate but as an essential mediator linking itaconate signalling to transcriptional reprogramming and tumour progression.

### The itaconate–HSPA8 axis activates the ATF4 pathway to upregulate *PSAT1* expression

**Fig. 6:**
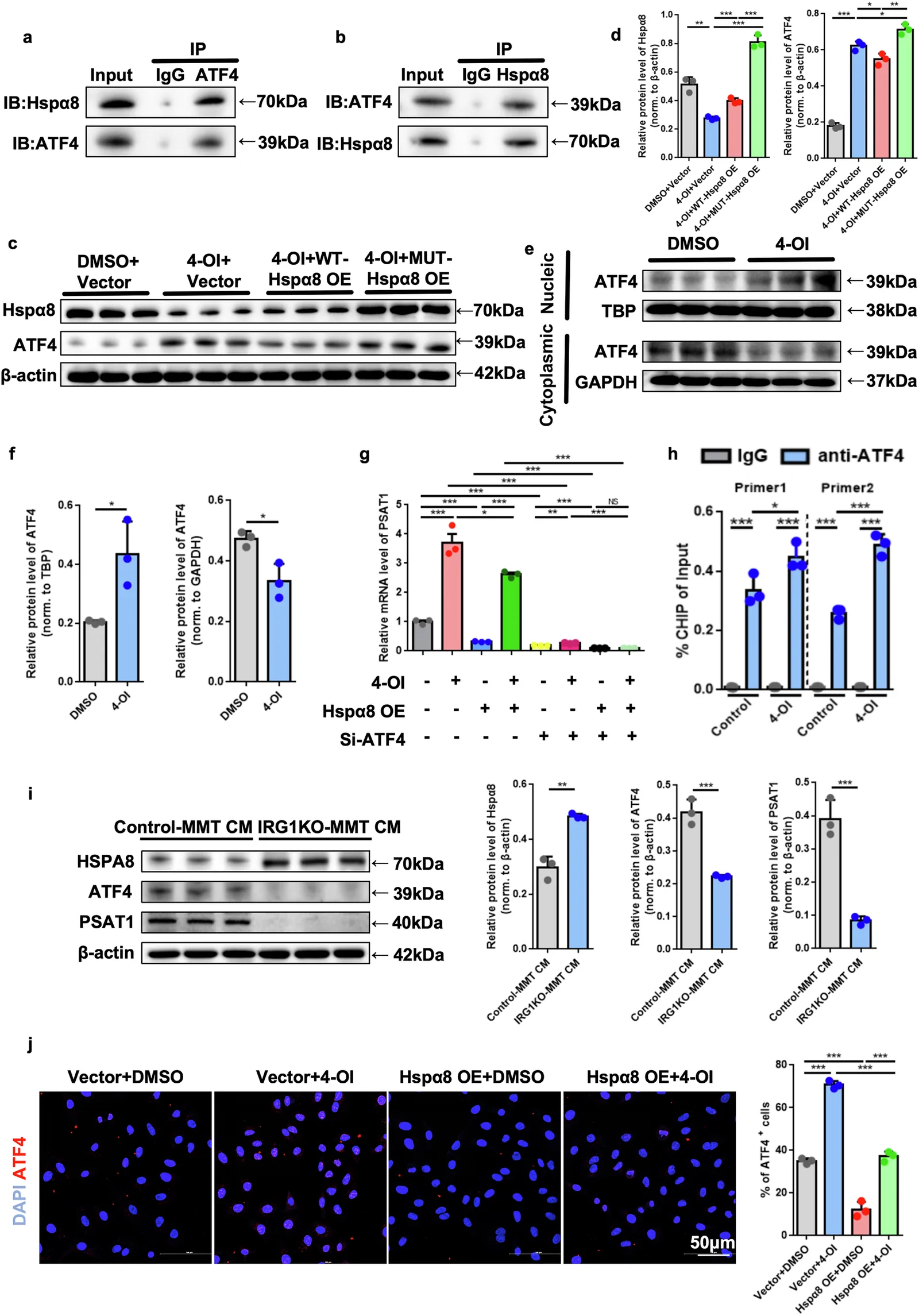
Itaconate-induced degradation of HSPA8 liberates ATF4 to transactivate *PSAT1.* Co-immunoprecipitation of *ATF4* and *HSPA8* (parts **a** and **b**). Western blotting for the expression of heat shock protein family A member 8 (HSPA8) and activated transcription factor 4 (ATF4) in A549 cells with 4-octyl itaconate (4-OI), overexpressed (OE) wild-type (WT) *HSPA8* or OE mutant (MUT) *HSPA8* (parts **c** and **d**). Western blotting of nuclear–cytoplasmic fractionation for the expression of ATF4 in A549 cells treated with 4-OI (parts**e** and **f**). The expression of *PSAT1* in cells with 4-OI, *HSPA8* OE or *ATF4* knockdown (si-ATF4) (part **g**). Chromatin immunoprecipitation analysis of *ATF4* occupancy at the *PSAT1* promoter (part **h**). Western blot analysis of HSPA8, ATF4 and phosphoserine aminotransferase 1 (PSAT1) protein levels in cells treated with macrophage-to-myofibroblast transition (MMT)-conditioned medium or *IRG1*-knockout MMT-conditioned medium (part **i**). Representative immunofluorescence images (part **j**, left panel) and mean fluorescence intensity (part **j**, right panel) of ATF4 in response to different treatments. Scale bar, 50 µm.

Given that itaconate-induced degradation of HSPA8 likely liberates client proteins normally held under chaperone restraint, it is likely that a transcription factor sequestered or stabilized by HSPA8 could be released, thereby gaining access to the nucleus to drive *PSAT1* expression. Among stress-responsive transcription factors regulated by molecular chaperones, ATF4, a master effector of the integrated stress response, is particularly compelling. The integrated stress response is the primary transcriptional programme activated in response to metabolic and proteotoxic perturbations, and ATF4 is well established to orchestrate amino acid metabolism, redox homeostasis and serine biosynthesis by directly binding to the promoters of key pathway genes, including *PSAT1* (ref. ^38^). Moreover, emerging evidence suggests that HSPA8 can modulate ATF4 stability or activity under stress conditions, positioning ATF4 as a strong candidate for the transcriptional mediator linking itaconate–HSPA8 signalling to *PSAT1* upregulation^39,40^. Hence, whether HSPA8 directly associates with ATF4 in lung cancer bone metastasis was assessed. Co-IP confirmed a direct physical interaction between endogenous HSPA8 and ATF4 in A549 cells. Immunoprecipitation with an anti-ATF4 antibody successfully pulled down HSPA8 (~70 kDa) (Fig. 6a), while reciprocal immunoprecipitation using an anti-HSPA8 antibody retrieved ATF4 (~39 kDa) (Fig. 6b). No signal was detected with control IgG, confirming the specificity of the interaction. These results demonstrate that HSPA8 forms a stable complex with ATF4, likely restraining its nuclear translocation. In vector-transfected cells treated with DMSO, HSPA8 exhibited robust expression, in contrast to the markedly lower levels of ATF4. Upon 4-OI treatment, HSPA8 protein was markedly reduced, accompanied by a concomitant increase in total ATF4. Overexpression of wild-type *HSPA8* counteracted 4-OI-mediated HSPA8 loss and suppressed ATF4 accumulation, whereas the ubiquitination-resistant *HSPA8* mutant remained stable and fully prevented ATF4 upregulation (Fig. 6c, d). These findings indicate that itaconate promotes the ubiquitin-dependent degradation of HSPA8, thereby relieving its inhibitory hold on ATF4 and enabling ATF4 stabilization and accumulation. Critically, subcellular fractionation followed by Western blotting revealed that 4-OI treatment significantly enhanced ATF4 nuclear translocation, an effect reversed by wild-type *HSPA8* overexpression but not by the *HSPA8* mutant (Fig. 6e, f). This confirms that HSPA8 degradation directly facilitates ATF4 nuclear import. To assess the functional consequences of this cascade, the transcription of *PSAT1* was examined. qRT-PCR showed that 4-OI alone induced *PSAT1* mRNA expression; this induction was attenuated by *HSPA8* overexpression and nearly abolished upon *ATF4* knockdown (Fig. 6g). Chromatin immunoprecipitation assays further demonstrated that ATF4 specifically binds to the PSAT1 promoter, as evidenced by significant enrichment with anti-ATF4 antibody but not control IgG (Fig. 6h). To investigate the downstream signalling pathways regulated by IRG1 in the context of MMT-mediated crosstalk, recipient cells were treated with conditioned medium collected from either control MMT cells or *IRG1-*knockout MMT cells. Western blot analysis revealed that the protein levels of HSPA8 were significantly upregulated, while the expression of ATF4 and PSAT1 was significantly downregulated following treatment with *IRG1-*knockout MMT-CM (Fig. 6i). In addition, immunofluorescence staining corroborated these findings, showing that HSPA8 overexpression retained ATF4 in the cytoplasm, whereas 4-OI promoted its nuclear accumulation (Fig. 6j). Together, these data establish a coherent metabolic–transcriptional axis: itaconate triggers the ubiquitin-mediated degradation of HSPA8, releasing ATF4 from cytosolic sequestration, promoting its nuclear translocation, enhancing its occupancy on the PSAT1 promoter and ultimately driving PSAT1 expression — thereby completing the itaconate–HSPA8–ATF4–PSAT1 signalling cascade.

### AAV-mediated *PSAT1* knockdown slows tumour progression

**Fig. 7:**
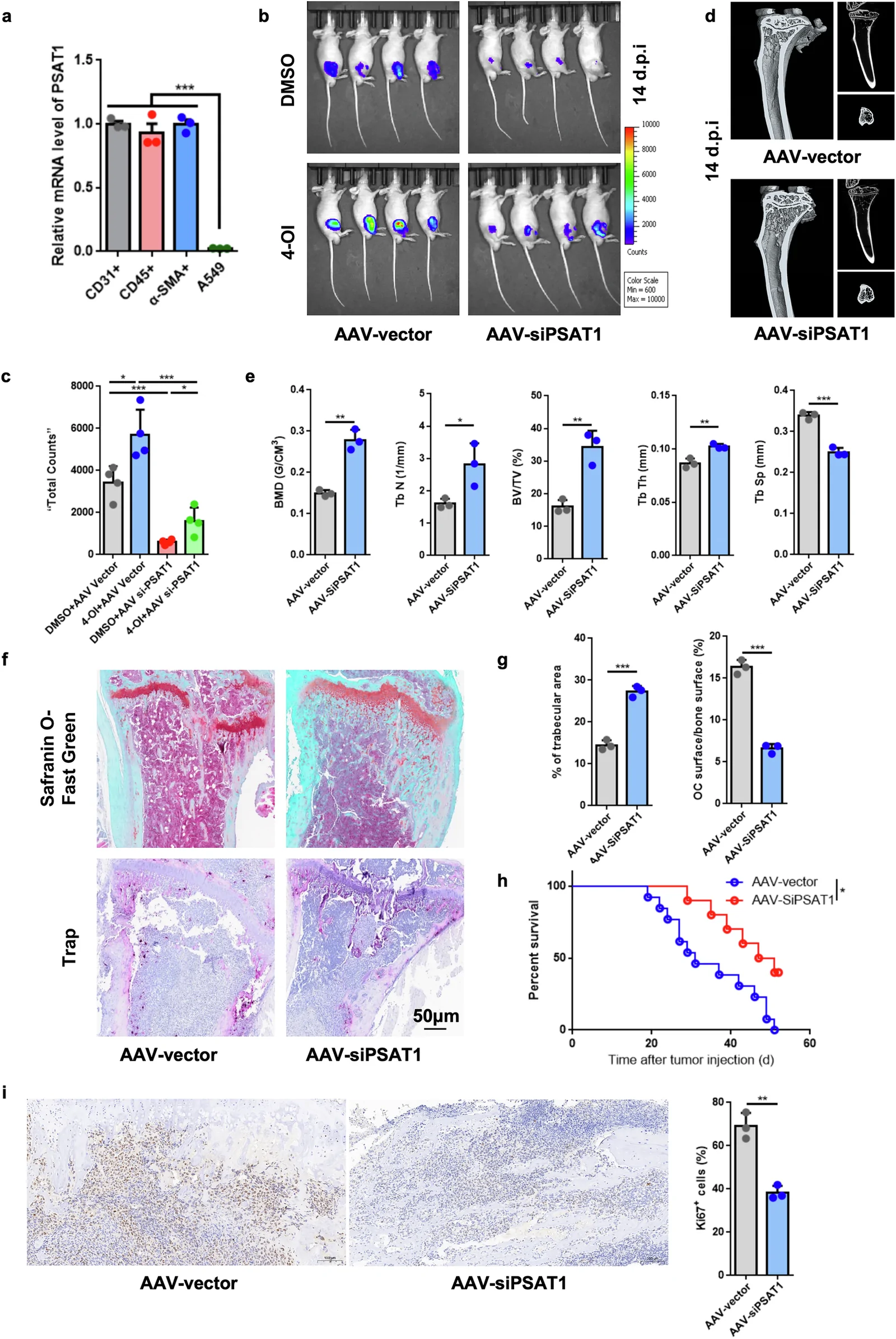
PSAT1 silencing reversed itaconate-mediated tumour and osteolytic progression. **a** Quantitative real-time PCR analysis of phosphoserine aminotransferase 1 (PSAT1) expression in fluorescence-activated cell sorting (FACS)-sorted cell populations. **b** Representative live bioluminescence images of luciferase-labelled A549 (A549-luc) tibial tumours treated with 4-octyl itaconate (4-OI) or AAV-siPSAT1. **c** Quantitative statistics of the bioluminescence signal intensity. **d** Micro-CT reconstruction of tibias treated with adeno-associated virus (AAV) control (AAV-vector) or AAV-siPSAT1. **e** Quantitative statistics of micro-CT. **f** Safranin O-Fast Green staining (upper panel) and TRAP staining (lower panel). Scale bar, 50 µm. **g** Quantitative statistics of part **f**. **h** Kaplan–Meier survival curve of A549-luc mice treated with AAV-vector or AAV-siPSAT1. **i** Ki67 staining. Scale bar, 100 µm.

To evaluate the therapeutic effect of PSAT1 silencing in vivo, A549-luc tumour-bearing mice were treated with adeno-associated virus (AAV) via intratumoural multi-point injection on day 3 post tumour implantation. Tumours were harvested on day 14 for subsequent analysis. To verify the cell-type specificity and efficiency of PSAT1 knockdown, tumour tissues were enzymatically dissociated into single-cell suspensions and subjected to FACS. Distinct cellular populations were identified using lineage markers, including CD31 for endothelial cells, CD45 for immune cells and αSMA for fibroblast-like cells, while A549 tumour cells were distinguished based on constitutive FITC labelling. qRT-PCR analysis of sorted populations revealed a significant reduction of PSAT1 expression specifically in A549 tumour cells in the AAV-siPSAT1 group compared with controls (Fig. 7a). In contrast, no significant changes in PSAT1 expression were observed in endothelial, immune or fibroblast populations, indicating minimal off-target transduction. This study has proved that itaconate was associated with HSPA8 degradation, promoting nuclear translocation of ATF4 and increasing transcription of *PSAT1*; thus, *PSAT1* may be a potential therapeutic target of this axis. To test this hypothesis, in vivo experiments were conducted where A549-luc subcutaneous tumour-bearing mice were randomly assigned to DMSO or 4-OI groups and received AAV-control or AAV-siPSAT1. In vivo imaging on day 21 revealed that bioluminescence was intensified in the 4-OI + AAV-control group compared with that in the DMSO + AAV-control group, whereas the signal was markedly reduced in the 4-OI + AAV-siPSAT1 group (Fig. 7b, c). Micro-CT revealed that 4-OI markedly increased the osteolytic volume in the tibial metastatic model, and this effect was completely blocked by AAV-siPSAT1 (Fig. 7d, e). Safranin O-Fast Green and TRAP staining of treated tibiae were performed for histometric evaluation (Fig. 7f, g). The mice treated with AAV-siPSAT1 displayed significantly greater Safranin O-positive red staining at the cartilage–bone junction and improved cortical continuity with Fast Green, indicating a preserved cartilage matrix and enhanced mineralization. Concurrently, the number of TRAP-positive osteoclasts (purple) was reduced, and the osteoclast density per bone surface was significantly lower, suggesting that osteoclastic differentiation was suppressed. Moreover, the median survival was extended to 50 days in the AAV-siPSAT1 group versus 30 days in the AAV-control group (Fig. 7h). Ki67 immunostaining of excised tumours revealed a significantly lower positive rate in AAV-siPSAT1-treated mice, further demonstrating that PSAT1 silencing profoundly inhibited tumour cell proliferation (Fig. 7i).

## Discussion

**Fig. 8:**
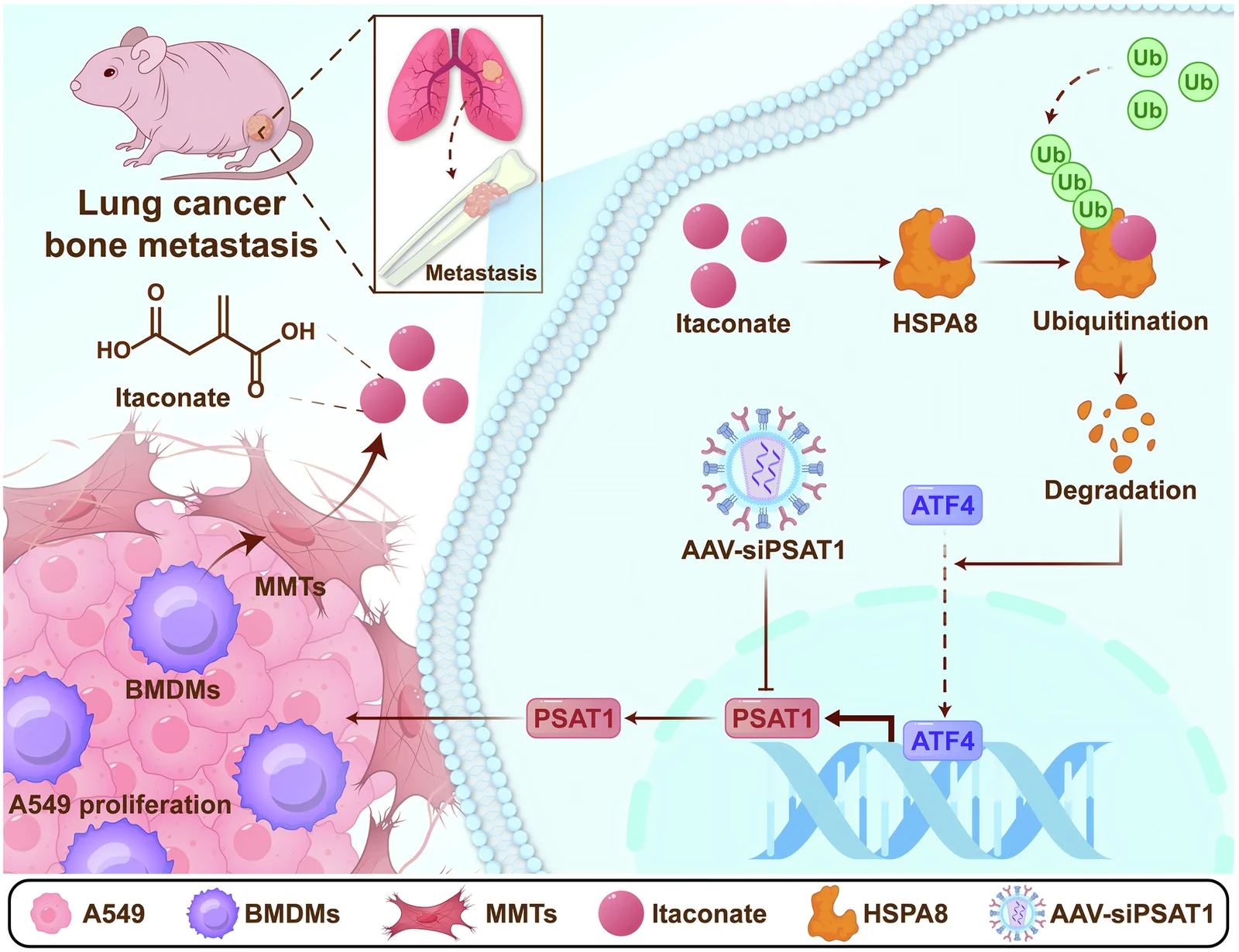
Schematic of the lung cancer bone metastatic microenvironment. Within a cortical bone defect, tumour-associated macrophages elongate, progressively acquiring spindle shapes and losing their rounded nuclei to undergo macrophage-myofibroblast transformation (MMT). These nascent MMT cells densely secrete itaconate, which is taken up by adjacent lung tumour cells. Intracellular itaconate promotes the ubiquitin–proteasome machinery, increasing heat shock protein family A member 8 (HSPA8) ubiquitination and thereby alleviating the inhibition of activated transcription factor 4 (ATF4) activity. ATF4 is translocated to the nucleus and binds to the phosphoserine aminotransferase 1 (PSAT1) promoter, and upregulates the expression of PSAT1. This ultimately amplifies tumour cell nuclear division and tumour proliferation in the bone destruction area. AAV, adeno-associated virus; BMDMs, bone marrow-derived macrophages.

This study shows solid in vivo evidence that the MMT is robust and closely related to lung cancer bone metastasis (Fig. 8). Lung cancer cell-conditioned medium, bone marrow mesenchymal stem cells and TGFβ were first used to reconstruct the tibial bone marrow niche. After co-culture, F4/80^+^ BMDMs underwent MMT and eventually transformed into αSMA^+^FAP^+^ CAF-like cells, with an efficiency similar to that reported in retinal, cardiac and renal fibrosis models^41–43^. In kidney, retina and other fibrotic disorders, the magnitude of MMT is directly correlated with disease severity. Tang et al. reported that renal tissues from patients with chronic kidney disease containing more than 20% CD68^+^αSMA^+^ cells were associated with a 3.8-fold greater risk of progression to end-stage renal disease within 3 years^41^. Chen et al. demonstrated that P2Y12 inhibition, which blocks MMT, reduced cortical collagen deposition by approximately 40%, indicating that MMT levels predict the rate of functional decline^42^. Similarly, Little et al. reported that, in neovascular age-related macular degeneration, each 10% increase in the proportion of MMT cells within subretinal membranes increased the probability of vision loss and was significantly associated with a poor response to anti-VEGF therapy^43^. In the present study, a tumour-to-macrophage ratio of 1:2 was employed in both in vitro and in vivo experiments. While tumour cells are typically the dominant population in established tumours, this experimental design was intended to model the early stages of tumour progression and metastatic niche formation, where stromal and immune components play a critical role. Accumulating evidence suggests that, during early metastatic seeding, particularly in bone metastasis, myeloid cells are rapidly recruited and can transiently outnumber tumour cells at the site of colonization^44,45^. These infiltrating cells contribute to the establishment of a permissive microenvironment by promoting extracellular matrix remodelling, immunosuppression and growth factor secretion^46,47^. Therefore, a relatively higher proportion of macrophage lineage cells in the co-injection model may better recapitulate this early microenvironmental context. Furthermore, selection of the 1:2 ratio was empirically supported by preliminary experiments, which demonstrated that this condition achieves an optimal balance between tumour establishment, observable stromal effects and experimental feasibility. Higher ratios (for example, 1:4) did not further improve interpretability, whereas lower ratios (for example, 1:1) resulted in less pronounced stromal contributions. Furthermore, MMT actively fuels itaconate release within lung cancer bone lesions, enlarges osteolytic areas and decreases survival, establishing MMT not only as a stromal reaction but also as a key cellular engine of metastatic progression and providing direct evidence that myeloid lineage plasticity promotes skeletal colonization. These results, which are consistent with those of previous studies, have shown that the presence and high expression of MMT are closely related to worse prognoses of the disease.

These results indicate that MMT represents a dynamic and continuous transition rather than a binary state. During this process, macrophages progressively downregulate F4/80 while acquiring myofibroblast-like features, resulting in a transitional population characterized by intermediate or low F4/80 expression. These cells may still be partially susceptible to anti-F4/80-mediated depletion, particularly at early stages of the transition. More importantly, the functional experiments demonstrate that IRG1 silencing exerts a significantly greater inhibitory effect in MMT cells compared with conventional macrophages, indicating that the tumour-promoting activity of IRG1–itaconate is markedly enhanced following MMT transition. These findings suggest that, although conventional macrophages contribute to the overall itaconate pool, MMT cells exhibit a higher functional dependency on IRG1-driven metabolic reprogramming. These data support a model in which both macrophages and MMT cells participate in shaping the tumour microenvironment but MMT-derived IRG1–itaconate acts as the predominant contributor to tumour progression and osteolytic remodelling. This highlights the importance of cellular state-specific metabolic regulation in determining the functional output of immunometabolic pathways.

While the present findings support a tumour-promoting role of the IRG1–itaconate axis in MMT and bone metastatic progression, accumulating evidence suggests that the biological functions of itaconate are highly context-dependent. Notably, several studies have reported anti-inflammatory and potentially antitumour properties of itaconate, primarily through inhibition of succinate dehydrogenase and activation of NRF2 signalling^48,49^. In this context, itaconate has been shown to restrain excessive immune activation and limit inflammatory damage. Conversely, emerging evidence indicates that itaconate can also exert pro-tumour effects within the tumour microenvironment. As highlighted in recent studies, itaconate-mediated metabolic reprogramming may contribute to immunosuppressive phenotypes in myeloid cells, thereby dampening antitumour immunity^50,51^. These seemingly contradictory observations underscore the dual and context-dependent nature of itaconate as an immunometabolic regulator. Importantly, the present study extends this framework by linking itaconate metabolism to macrophage phenotypic plasticity, specifically the acquisition of myofibroblast-like features during MMT. This suggests that, beyond its classical role in inflammatory regulation, itaconate may participate in cell fate transitions that reshape the tumour stroma. In the context of bone metastasis, such metabolic reprogramming likely operates in concert with other well-established pathways. Tumour progression and metastatic niche formation are known to involve coordinated alterations in glycolysis, hypoxia signalling and TCA cycle-associated metabolites^52–54^. These interconnected metabolic adaptations collectively contribute to extracellular matrix remodelling, immune modulation and stromal activation within metastatic sites.

Mechanistically, MMT leads to a localized metabolic niche by continuously releasing itaconate. Quantitative mass spectrometry revealed that the level of intracellular itaconate in MMT cells was much greater than that in resident macrophages, tumour cells, or infiltrating lymphocytes and neutrophils within the same lesion, indicating a cell-type-restricted distribution that qualifies itaconate as a CAF subset-specific metabolic signature^55,56^. In vivo, both tumour and peripheral blood showed significantly elevated itaconate levels only when the transformation had completed, whereas the number of BMDMs remained low, demonstrating that converted CAFs acquire a durable metabolic memory that perpetually fuels the bone lesion. To test whether the efflux was functionally decisive, mice bearing intratibial A549-luc tumours were treated with either the cell-permeable itaconate derivative 4-OI or the membrane-transport inhibitor citraconate. Micro-CT quantification revealed that 4-OI reconstituted the osteolytic area within 14 days, doubled the tumour burden and shortened median survival, indicating that itaconate alone suffices to recapitulate the destructive phenotype. Conversely, citraconate blocked 4-OI uptake and completely abolished its prolytic effects, confirming that secreted itaconate must be reimported to operate. Collectively, the data establish itaconate as a molecular switch that couples immunometabolism to stromal remodelling and identify its selective interception as a tractable strategy to break the vicious osteolytic–proliferative cycle in lung cancer bone metastases.

With respect to effectors, drug affinity responsive target stability assays revealed that itaconate dose-dependently shields HSPA8 from pronase digestion, indicating high-affinity binding. Using cross-linking mass spectrometry, the contact interface was mapped to the SBD subdomain of HSPA8 and to the 2,3-dicarboxy double bond of itaconate, confirming a specific metabolite–protein interaction that licences TRIM21-mediated K48-linked ubiquitination and proteasomal degradation of HSPA8 (ref.^57^). Loss of the chaperone liberates ATF4, which is translocated to the nucleus, where it occupies the AARE elements of the PSAT1 promoter and increases serine glycine one-carbon flux^58–60^. The resulting expansions of nucleotide and glutathione pools endow cancer cells with a proliferative advantage in glucose-poor, reactive oxygen species-rich cortical bone^61,62^. Furthermore, treatment with AAV-siPSAT1 permanently silenced PSAT1 in metastatic lesions and completely inhibited both 4-OI-driven tumour growth and osteolysis. Therefore, PSAT1 is a treatable target in the itaconate–HSPA8–ATF4 axis, in which immune metabolites in turn reshape protein stability and metabolic enzyme expressions to alter bone metastases in lung cancer.

In conclusion, this study elucidated a complete signalling chain of MMT, itaconate, HSPA8 ubiquitination, ATF4 nuclear translocation and PSAT1 transcription that promotes lung cancer bone metastases. Furthermore, it was demonstrated, in vivo, that a single systemic dose of AAV-siPSAT1 exacerbates this cascade, decreases the number of osteolytic lesions and extends survival. By positioning MMT as a metabolic and stromal crossroad, the present study offers a series of intervention strategies such as blocking IRG1–itaconate production, stabilizing HSPA8 to prevent the release of ATF4, or silencing PSAT1 directly. Future work should validate the efficacy of this axis in large patient cohorts and advance combined metabolic–immune regimens into the clinic, developing a precise and low-toxicity therapeutic avenue for individuals afflicted by lung cancer bone metastasis.

## Supplementary information
