# Supplementary material for: Macrophage-to-myofibroblast transition-derived itaconate promotes bone metastasis in lung cancer through targeting of HSPA8
Source: Exp Mol Med. 2026 Aug 5;58(8):2670–87. doi: 10.1038/s12276-026-01799-9 (PMC13538512; doi:10.1038/s12276-026-01799-9)
Supplement: Supplementary file 1 [file 12276_2026_1799_MOESM1_ESM.pdf]

Supplementary materials

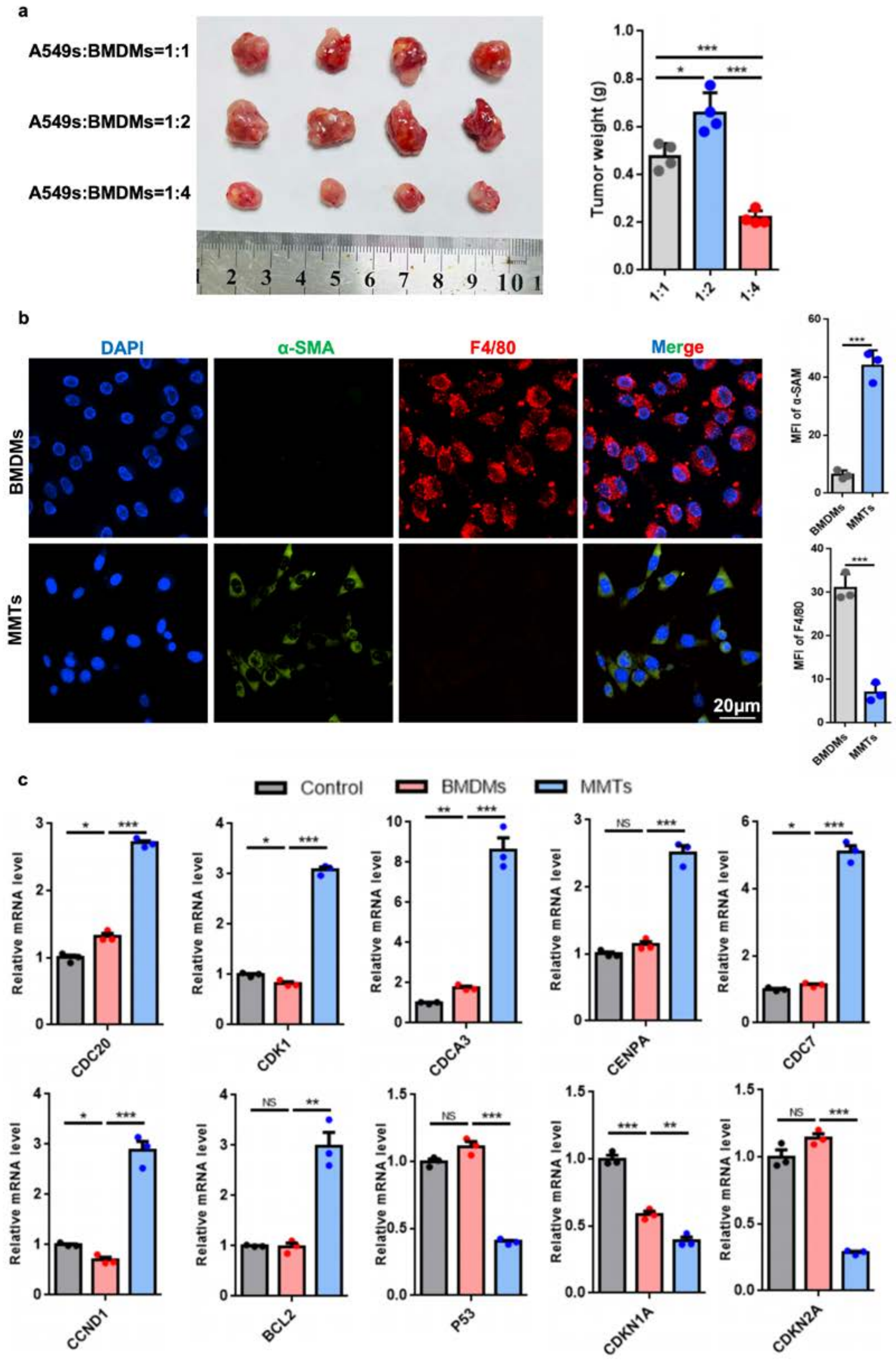

Supplementary Fig. 1. Characterization and proliferative/apoptotic profile of MMTs.

(a) Tumor growth analysis of subcutaneous xenografts established with different A549:BMDM ratios (1:1, 1:2, and 1:4; tumor cells:macrophages). (b) Representative immunofluorescence images (left panel) and quantification of the mean fluorescence

intensities (right panel) of α-SMA (green) and F4/80 (red). Scale bar, 50 μm (c) RT–PCR analysis of the mRNA levels of *CDC20*, *CDK1*, *CDCA3*, *CENPA*, *CDC7*, *CCND1*, *P53*, *BCL2*, *CDKN1A* and *CDKN2A*.

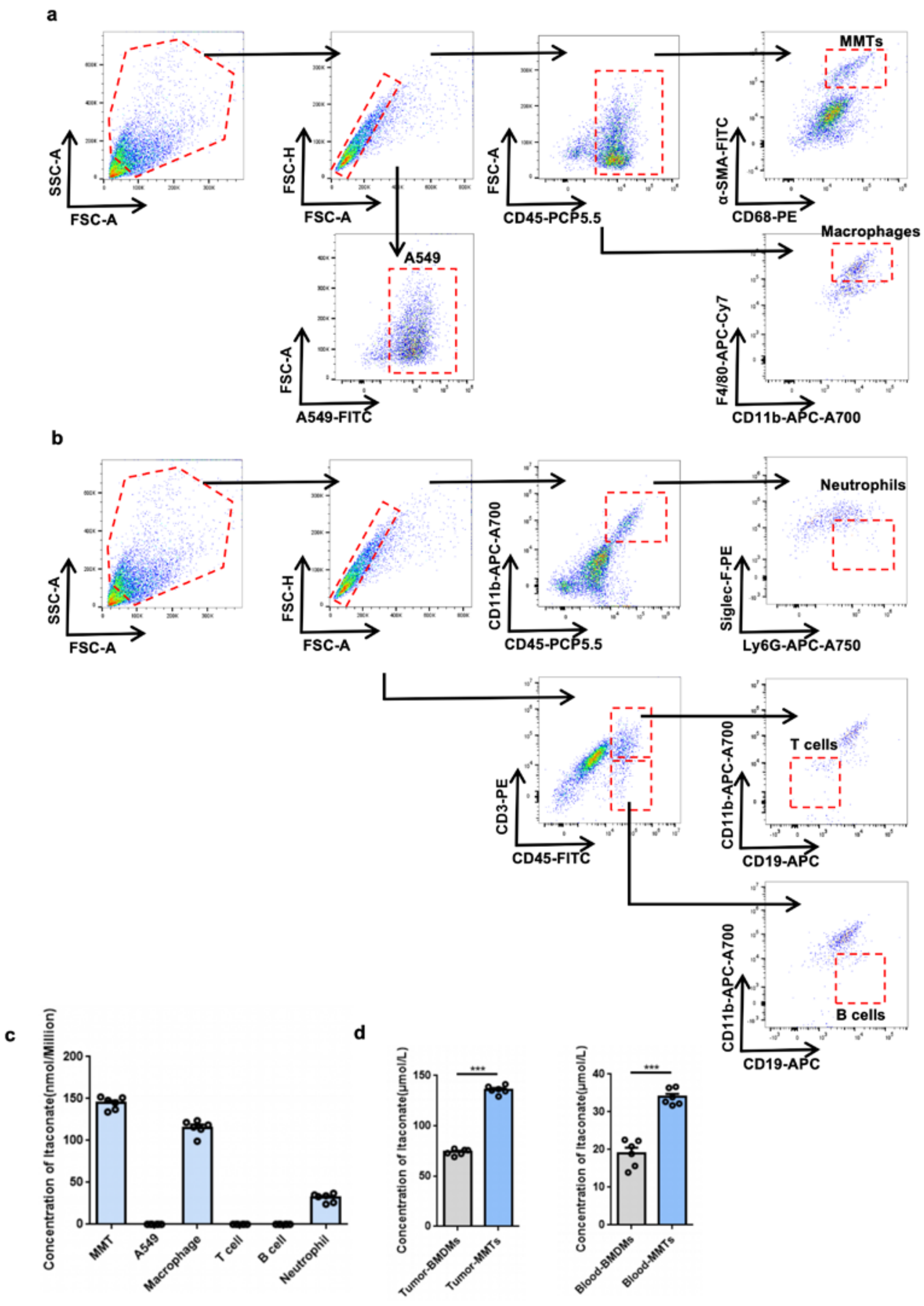

Supplementary Fig. 2. Cell type–specific distribution of itaconate within the tumor microenvironment.

(a) Representative flow cytometry gating strategy for isolation of distinct cellular populations from dissociated tumor tissues. Single-cell suspensions were sequentially gated to identify macrophages, MMT cells, neutrophils, tumor cells, T cells, and B cells based on lineage-specific markers. (b) Quantification of sorted cell populations showing the relative abundance of each subset within the

tumor microenvironment. (c) Intracellular itaconate levels in FACS-sorted cell populations. Equal numbers of each cell type were lysed and subjected to ELISA analysis. (d) Itaconate concentration in tumor tissues and peripheral blood from mice co-injected with A549 + BMDMs or A549 + MMTs.

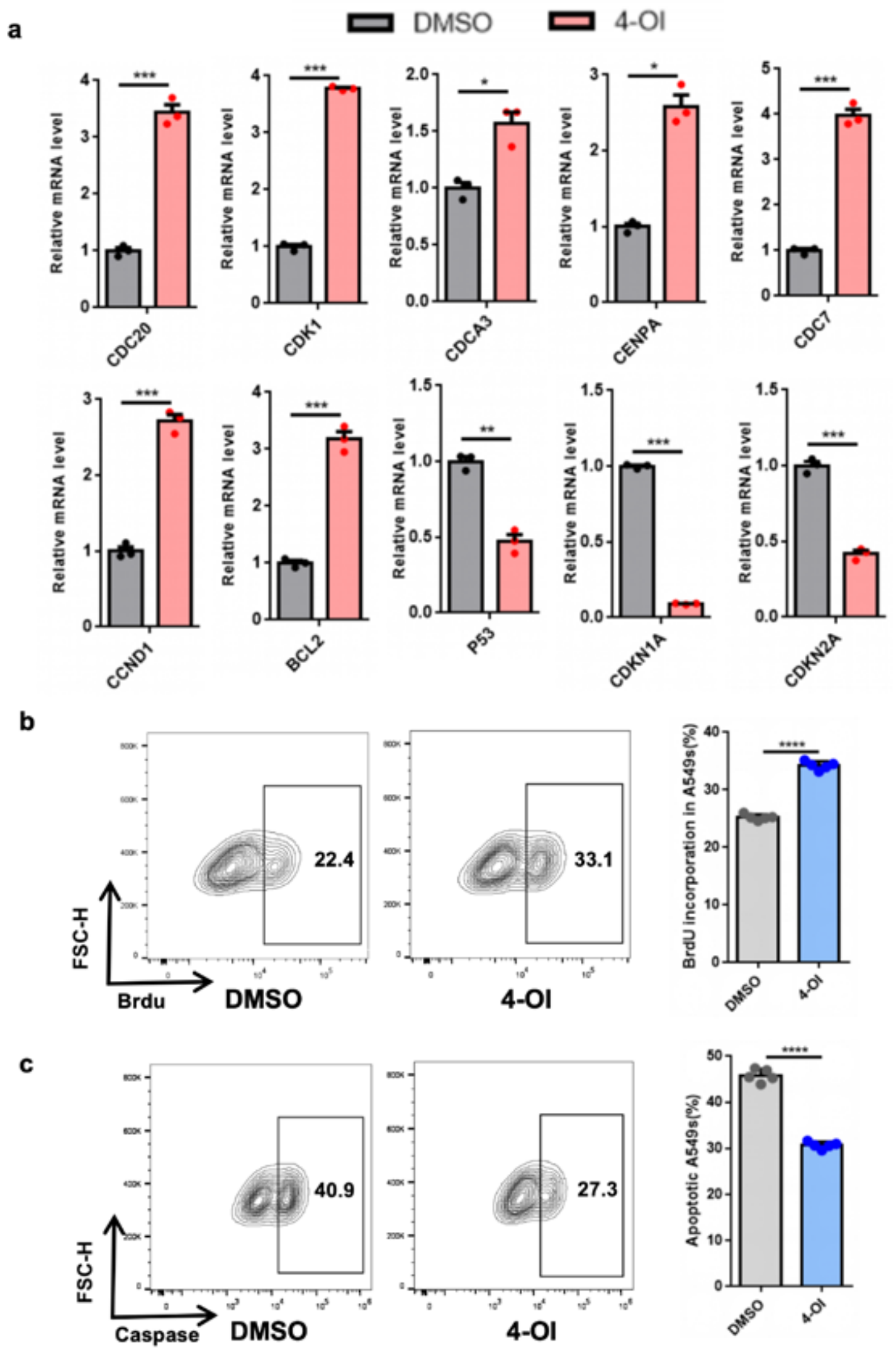

Supplementary Fig. 3. Exogenous itaconate promotes proliferation and suppresses apoptosis in lung cancer cells.

(a) qRT–PCR analysis of the mRNA levels of *CDC20*, *CDK1*, *CDCA3*, *CENPA*, *CDC7*, *CCND1*, *P53*, *BCL2*, *CDKN1A* and *CDKN2A* in A549 cells treated with 4-OI or DMSO. (b) Representative flow cytometry images (left panel) and quantification (right panel) of BrdU in A549 cells treated with 4-OI or DMSO. (c) Representative flow cytometry images (left panel) and quantification (right panel) of caspase in A549 cells treated with 4-OI or DMSO.

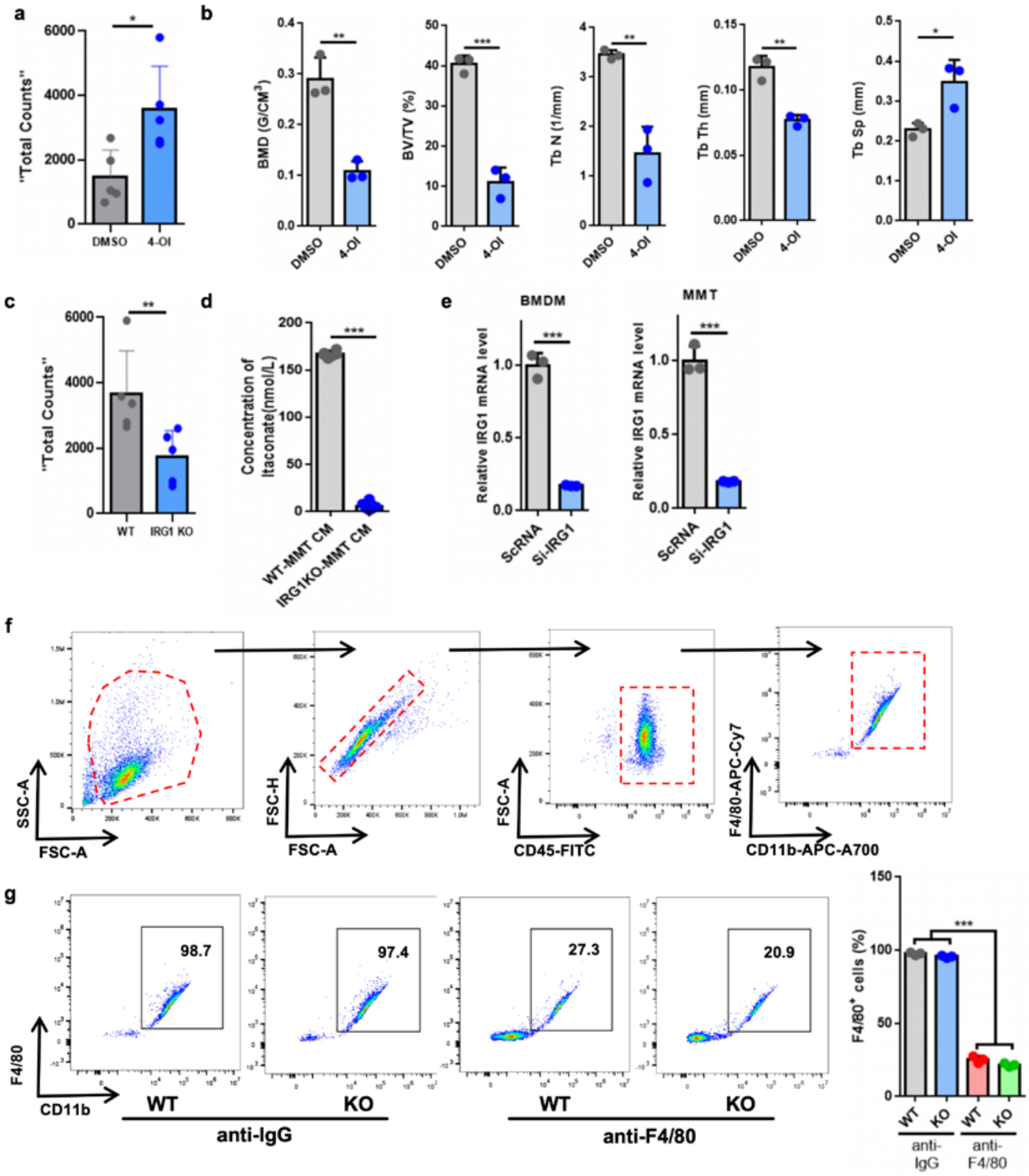

Supplementary Fig. 4. Oncogenic effects of 4-OI and *in vivo* macrophage neutralization.

(a) Quantification of the bioluminescence signal in A549-luc tumor-bearing mice treated with or without 4-OI. (b) Quantification of micro-CT, including BMD, BV/TV, Tb.N, Tb.Th and Tb.Sp. (c) Quantification of bioluminescence images of WT and *IRG1*-KO mice. (d) Intracellular itaconate levels in WT-MMT and IRG1 KO-MMT groups. Quantitative analysis of itaconate concentration in MMT cells derived from WT and *Irg1*-KO mice. IRG1 deficiency resulted in a marked reduction in itaconate levels compared with the WT-MMT group. (e) Relative IRG1 mRNA expression following siRNA-mediated knockdown in BMDMs and MMT cells. (f) Gating strategy for flow cytometry of neutralized macrophages. (g) Representative flow cytometry images (left panel) and quantification (right panel) of macrophage depletion.

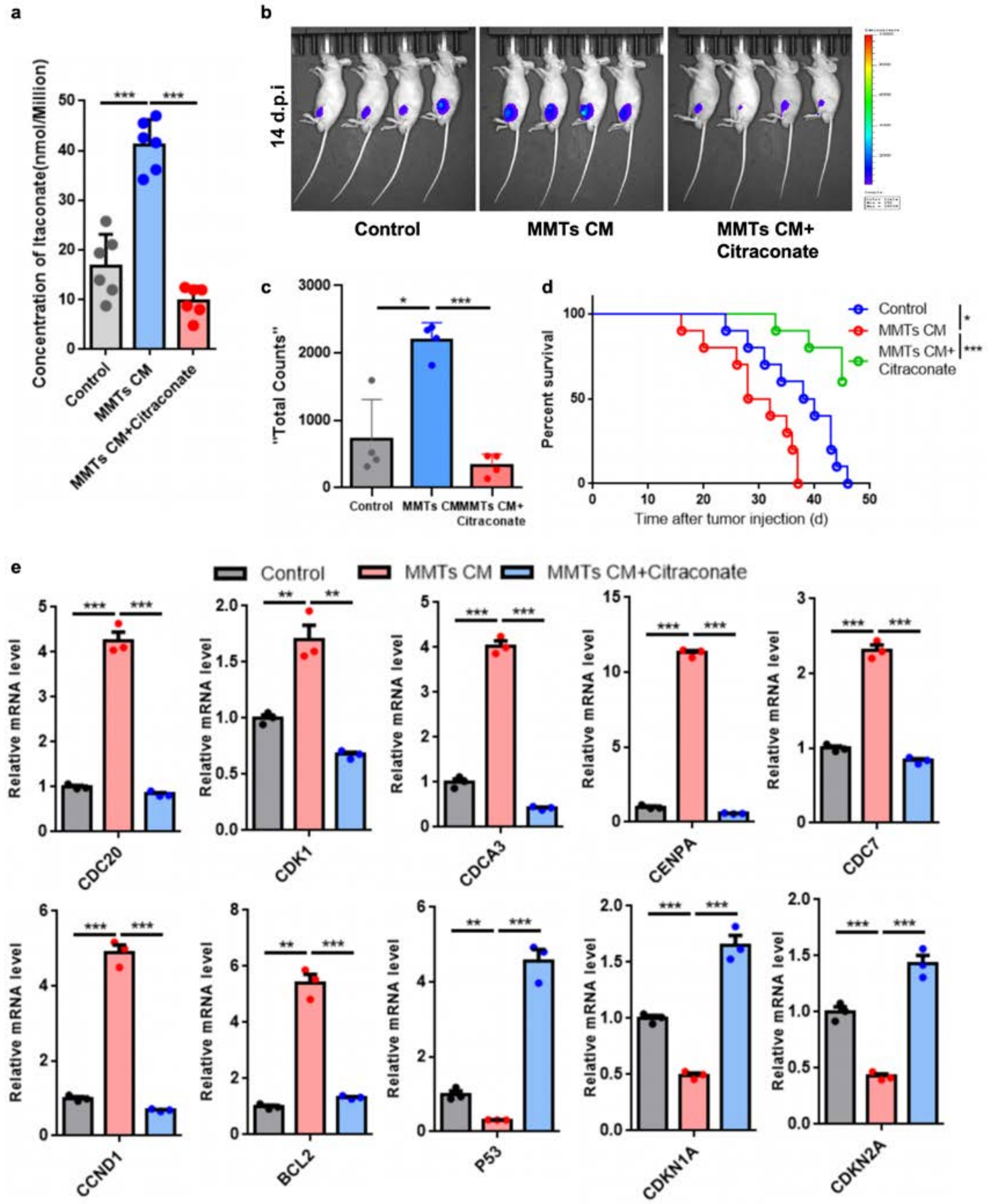

Supplementary Fig. 5. Citraconate blunts MMT-derived itaconate and restrains tumor growth. (a) Itaconate concentration in conditioned medium from MMT cells treated with or without citraconate *in vitro*. (b, c) Representative bioluminescence images and quantification of A549-luc-bearing mice. A549-luc cells were preconditioned with indicated CM for 48 hours *in vitro*, then injected into tibiae for bone metastasis modeling. (d) Survival times of A549-luc tumor-bearing mice treated with control CM, MMT-CM or MMT-CM supplemented with citraconate. (e) The mRNA expressions of apoptosis- and proliferation-related genes.

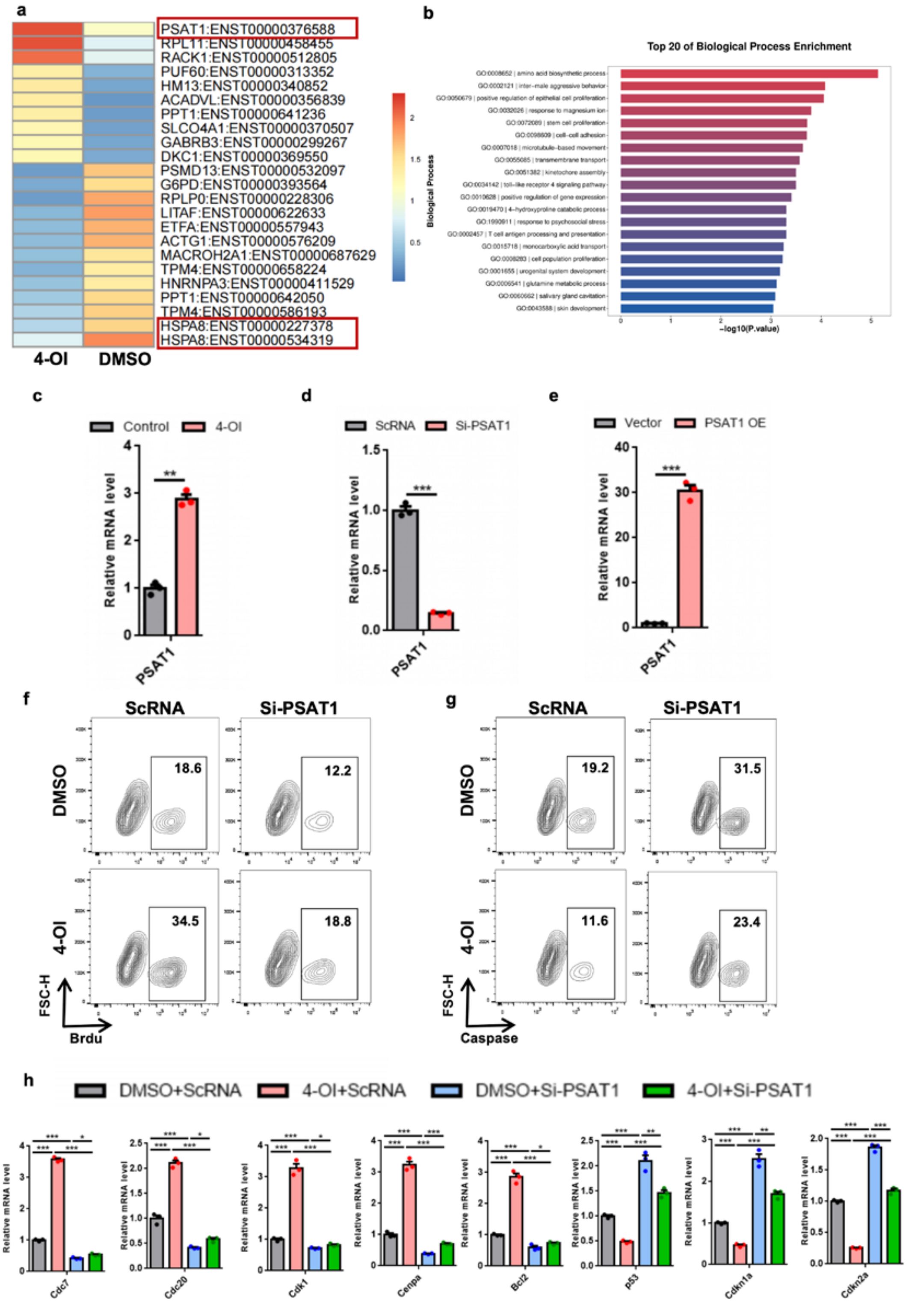

Supplementary Fig. 6. Itaconate-induced PSAT1 reprograms lung-cancer cell cycles and survival. (a) Heatmap of the RNA-seq data. (b) KEGG enrichment. (c) Expression of PSAT1 mRNA in A549 cells treated with 4-OI. (D-E) Expressions of PSAT1 mRNA in lung cancer cells with PSAT1 knockdown (d) or overexpression (e). (f) Representative flow cytometry images (left panel) and quantification (right panel) of BrdU in A549 cells treated with 4-OI or si-PSAT1. (g) Representative flow cytometry images (left panel) and quantification (right panel) of caspase in A549 cells treated with 4-OI or si-PSAT1. (h) RT–PCR

detection of the mRNA expressions of cell cycle/apoptosis genes in A549 cells treated with 4-OI or si-PSAT1.

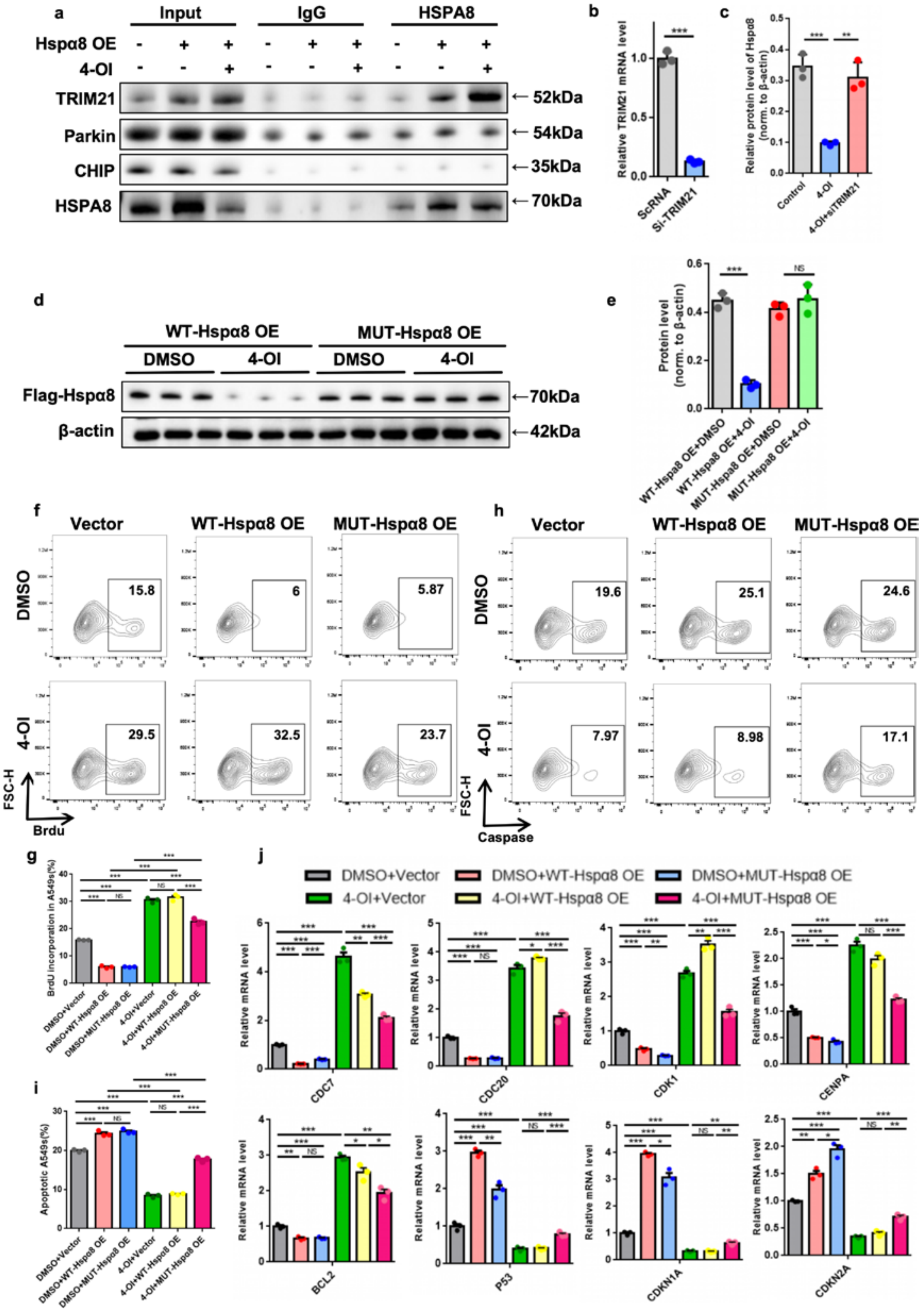

Supplementary Fig. 7. Disruption of the itaconate–HSPA8 interaction abolishes oncogenic signaling.

(a) Co-immunoprecipitation analysis of HSPA8 with candidate E3 ubiquitin ligases in A549

cells treated with 4-OI or DMSO for 24 hours. (b) qRT-PCR analysis of relative TRIM21 mRNA expression levels after siRNA-mediated TRIM21 knockdown. (c) Quantitative analysis of Western blot showing the effect of TRIM21 knockdown on Hspa8 protein levels under 4-OI treatment. (d) Western blotting revealed that when HSPA8 was overexpressed, 4-OI significantly decreased the protein level of WT-HSPA8, whereas MUT-HSPA8 expressions were not affected by 4-OI. (e) Relative Flag-HSPA8 abundances normalized to that of β-actin. (f, g) Representative flow cytometry images (left panel) and quantification (right panel) of BrdU in A549 cells treated with 4-OI, WT-*HSPA8*-OE or MUT-*HSPA8*-OE. (h, i) Representative flowcytometry images (left panel) and quantification (right panel) of caspase in A549 cells treated with 4-OI, WT-*HSPA8*-OE or MUT-*HSPA8*-OE. (j) The mRNA expressions of cell cycle/apoptosis genes in A549 cells treated with 4-OI, WT-*HSPA8*-OE or MUT-*HSPA8*-OE.

Supplementary Table 1. Detailed clinical and molecular information.

NO.	Age (years)	Sex	Smoking history	Histological subtype	Metastasis site	The expression of α-SMA	Survival rate (mouths)
1	71	Male	30 years	LUAD	Bone	High	10
2	54	Male	No	LUAD	Bone	High	13
3	69	Male	No	LUAD	Bone	High	13
4	38	Male	No	LUAD	Bone	High	7
5	82	Male	30 years	LUAD	Bone	High	12
6	73	Female	No	LUAD	Bone	High	15
7	66	Male	No	LUAD	Bone	High	8
8	46	Male	No	LUAD	Bone	High	6
9	68	Male	No	LUAD	Bone	High	7
10	55	Male	No	LUAD	Bone	High	14
11	66	Male	No	LUAD	Bone	High	22
12	64	Female	No	LUAD	Bone	High	27
13	69	Female	No	LUAD	Bone	High	31
14	73	Female	No	LUAD	Bone	High	33
15	72	Male	No	LUAD	Bone	High	15
16	70	Male	30 years	LUAD	Bone	High	16
17	74	Female	No	LUAD	Bone	High	13
18	53	Male	No	LUAD	Bone	High	15
19	49	Female	No	LUAD	Bone	High	11
20	60	Male	No	LUAD	Bone	High	8
21	56	Female	No	LUAD	Bone	High	16
22	39	Male	No	LUAD	Bone	High	9
23	47	Male	20 years	LUAD	Bone	High	10
24	48	Male	No	LUAD	Bone	High	2
25	67	Female	No	LUAD	Bone	High	3
26	56	Female	No	LUAD	Bone	High	15
27	47	Female	No	LUAD	Bone	High	9
28	58	Male	No	LUAD	Bone	High	26
29	68	Male	No	LUAD	Bone	High	24
30	57	Male	No	LUAD	Bone	High	22
31	64	Male	No	LUAD	Bone	High	20
32	70	Male	50 years	LUAD	Bone	High	21
33	63	Female	No	LUAD	Bone	High	17
34	70	Male	No	LUAD	Bone	High	11
35	70	Female	No	LUAD	Bone	High	9
36	63	Male	No	LUAD	Bone	High	7
37	61	Male	No	LUAD	Bone	High	7
38	61	Male	No	LUAD	Bone	High	13

39	70	Male	40 years	LUAD	Bone	High	11
40	69	Male	No	LUAD	Bone	High	10
41	66	Female	No	LUAD	Bone	High	21
42	63	Female	No	LUAD	Bone	High	11
43	66	Female	No	LUAD	Bone	High	16
44	72	Male	30 years	LUAD	Bone	Low	5
45	45	Male	No	LUAD	Bone	Low	7
46	70	Male	No	LUAD	Bone	Low	7
47	66	Male	No	LUAD	Bone	Low	19
48	69	Male	No	LUAD	Bone	Low	4
49	56	Male	No	LUAD	Bone	Low	3
50	54	Male	No	LUAD	Bone	Low	16
51	52	Male	No	LUAD	Bone	Low	11
52	69	Male	No	LUAD	Bone	Low	8
53	50	Female	No	LUAD	Bone	Low	15
54	73	Male	No	LUAD	Bone	Low	32
55	64	Female	No	LUAD	Bone	Low	25
56	72	Male	30 years	LUAD	Bone	Low	33
57	63	Male	No	LUAD	Bone	Low	34
58	64	Male	No	LUAD	Bone	Low	24
59	59	Male	No	LUAD	Bone	Low	14
60	37	Male	No	LUAD	Bone	Low	22
61	63	Male	No	LUAD	Bone	Low	16
62	65	Female	No	LUAD	Bone	Low	18
63	60	Male	No	LUAD	Bone	Low	36
64	68	Female	No	LUAD	Bone	Low	33
65	56	Male	No	LUAD	Bone	Low	14
66	58	Female	No	LUAD	Bone	Low	17
67	58	Male	30 years	LUAD	Bone	Low	26
68	62	Male	No	LUAD	Bone	Low	22
69	53	Male	30 years	LUAD+ LUSC	Bone	Low	18
70	54	Female	No	LUAD	Bone	Low	10
71	55	Male	No	LUAD+ LUSC	Bone	Low	4
72	63	Male	No	LUAD	Bone	Low	31
73	71	Male	No	LUAD	Bone	Low	26
74	61	Male	20 years	LUAD	Bone	Low	25
75	58	Female	No	LUAD	Bone	Low	17
76	59	Female	No	LUAD	Bone	Low	18
77	64	Male	40 years	LUSC	Bone	Low	20
78	63	Female	No	LUAD	Bone	Low	11
79	56	Male	No	LUAD	Bone	Low	13

80	60	Female	No	LUAD	Bone	Low	31
81	55	Male	No	LUAD	Bone	Low	26
82	61	Male	No	LUAD	Bone	Low	22

Supplementary Table 2. Reagent and resource in this study.

REAGENT or RESOURCE	SOURCE	IDENTIFIER
Antibodies		
Alexa Fluor®488 anti-mouse CD45 Antibody	Biolegend	Clone#: 30-F11;<br>Cat#103122;<br>RRID: AB_493531
Alexa Fluor® 700 anti-mouse/human CD11b Antibody	Biolegend	Clone#: M1/70;<br>Cat#101222;<br>RRID: AB_493705
Alexa Fluor® 700 anti-mouse CD11c Antibody	Biolegend	Clone#: N418;<br>Cat#117320;<br>RRID: AB_528736
APC/Cyanine7 anti-mouse F4/80 Recombinant Antibody	Biolegend	Clone#: QA17A29;<br>Cat#157316;<br>RRID: AB_2894638
PE anti-BrdU Antibody	Biolegend	Clone#: 3D4;<br>Cat#364116;<br>RRID: AB_2814317
PerCP/Cyanine5.5 anti-mouse CD45 Antibody	Biolegend	Clone#: S18009F;<br>Cat#157207;<br>RRID: AB_2860727
PE anti-mouse CD68 Antibody	Biolegend	Clone#: FA-11;<br>Cat#137013;<br>RRID: AB_10613469
Alexa Fluor® 488 anti-α Smooth Muscle Actin Antibody	Biolegend	Clone#: 1A4;<br>Cat#614853;<br>RRID: AB_3662308
APC/Cyanine7 anti-mouse Ly-6G Antibody	Biolegend	Clone#: 1A8;<br>Cat#127623;<br>RRID: AB_10645331
PE anti-mouse CD170 (Siglec-F) Antibody	Biolegend	Clone#: S17007L;<br>Cat#155505;<br>RRID: AB_2750234
PerCP/Cyanine5.5 anti-mouse CD45 Antibody	Biolegend	Clone#: S18009F;<br>Cat#157207;<br>RRID: AB_2860727
PE anti-mouse CD68 Antibody	Biolegend	Clone#: FA-11;<br>Cat#137013;<br>RRID: AB_10613469

Alexa Fluor® 488 anti-α Smooth Muscle Actin Antibody	Biolegend	Clone#: 1A4;<br>Cat#614853;<br>RRID: AB_3662308
APC/Cyanine7 anti-mouse Ly-6G Antibody	Biolegend	Clone#: 1A8;<br>Cat#127623;<br>RRID: AB_10645331
PE anti-mouse CD170 (Siglec-F) Antibody	Biolegend	Clone#: S17007L;<br>Cat#155505;<br>RRID: AB_2750234
ATF-4 (D4B8) Rabbit Monoclonal Antibody	Cell Signaling Technology	Clone#: D4B8;<br>Cat#11815;<br>RRID: AB_2616025
Anti-Ubiquitin Antibody	ABCAM	Clone#: EPR8830;<br>Cat#ab134953;<br>RRID: AB_2801561
Anti-6X His tag® Antibody	ABCAM	Clone#: HIS.H8;<br>Cat#ab18184;<br>RRID: AB_444306
Anti-DDDDK tag (Binds to FLAG® tag sequence) antibody	ABCAM	Clone#: EPR20018-251;<br>Cat#ab205606;<br>RRID: AB_2916341
Anti-HSPA8 Antibody	ABCAM	Clone#: N27F3-4;<br>Cat#ab47454<br>RRID: AB_10219262
Anti-Ki67 antibody	ABCAM	Clone#: SP6;<br>Cat#ab16667;<br>RRID: AB_302459
F4/80 Antibody	Affinity Biosciences	Cat#DF2789;<br>RRID: AB_2839995
FAP Antibody	Affinity Biosciences	Cat#AF5344;<br>RRID: AB_2837829
VEGFA Antibody	Affinity Biosciences	Cat#AF5131;<br>RRID: AB_2837617
alpha-SMA Antibody	Affinity Biosciences	Cat#AF1032;<br>RRID: AB_2835329
beta Actin Antibody	Affinity Biosciences	Cat#AF7018;<br>RRID: AB_2839420
GAPDH Antibody	Affinity Biosciences	Cat#AF7021;<br>RRID: AB_2839421
InvivoMAb anti-mouse F4/80	BioXcell	Clone#: CI:A3-1;<br>Cat#BE0206;<br>RRID: AB_10949019

InvivoMAb rat IgG2b isotype control	BioXcell	Clone#: LTF-2;<br>Cat#BE0090;<br>RRID: AB_1107780
Alexa Fluor® 488 AffiniPure Donkey Anti-Mouse IgG (H+L)	Jackson ImmunoResearch	Code: 715-545-151;<br>RRID: AB_2341099
Cy™3 AffiniPure Donkey Anti-Mouse IgG (H+L)	Jackson ImmunoResearch	Code: 715-165-150;<br>RRID: AB_2340813
Alexa Fluor® 488 AffiniPure Donkey Anti-Rabbit IgG (H+L)	Jackson ImmunoResearch	Code: 711-545-152;<br>RRID: AB_2313584
Horseradish enzyme labeled goat anti-rabbit IgG (H+L)	ZSGB-Bio	Cat#ZB-2301;<br>RRID: AB_2747412
Bacterial and virus strains		
HBAAV2/5-hTERT-h-siPSAT1-3xflag-ZsGreen	HANBIO (Shanghai, China)	N/A
Chemicals, peptides, and recombinant proteins		
Recombinant Human TGF beta 1 Protein	SinoBiological	Cat#10804-H08H1
Cycloheximide	MedChemExpress	Cat#HY-12320
MG-132	MedChemExpress	Cat#HY-13259
Tissue TeK O.C.T. Compound	SAKURA	Cat#4583
INVI DNA RNA Transfection Reagent	Invigentech	Cat#IV1216075
Critical commercial assays		
Phase-Flow™ FITC BrdU Kit	Biolegend	Cat#370704
Vybrant™ FAM Caspase-3 and -7 Assay Kit	Invitrogen	Cat#V35118
TRAP staining solution Kit	Wako	Cat#294-67001
Human itaconic acid ELISA Kit	MEIMIAN	Cat#MM-927908O1
PrimeScript™ RT Master Mix	Takara	Cat#RR036A
TB Green® Premix Ex Taq™ II	Takara	Cat#RR820A
Nuclear and Cytoplasmic Protein Extraction Kit	Beyotime	Cat#P0027
Experimental models: Cell lines		
A549 cells	Zhang's laboratory	
LLC cells	Zhang's laboratory	
Experimental models: Organisms/strains		
BALB/c-nu/nu nude mice	Guangdong Medical Laboratory Animal Center	N/A
Mouse: C57BL/6NCya-Acod1em1/Cya	Cyagen Bio-sciences Inc. (Suzhou, China)	Cat#S-KO-02679
Oligonucleotides		
Genotyping primer: Acod1 Forward: ACCGAGGATCGCTTAGCCTG	Cyagen Bio-sciences Inc. (Suzhou, China)	N/A

Genotyping primer: Acod1 Reverse: CAGAAGTCCGATTAGACACCAAAGC	Cyagen Bio-sciences Inc. (Suzhou, China)	N/A
qPCR Primer: F4/80 Forward: GTGCCATCATTGCGGGATTC	Primer Bank	N/A
qPCR Primer: F4/80 Reverse: AAGAGCATCACTGCCTCCAC	Primer Bank	N/A
qPCR Primer: VEGF Forward: CTGCTCTCTTGGGTGCACTG	Primer Bank	N/A
qPCR Primer: VEGF Reverse: GCAGCCTGGGACCACTTG	Primer Bank	N/A
qPCR Primer: FAP Forward: CATATGGGGCTGGTCCTACG	Primer Bank	N/A
qPCR Primer: FAP Reverse: AGCCACTGCTATGCCACATT	Primer Bank	N/A
qPCR Primer: alpha-SMA Forward: GCCATCTTTCATTGGGATGGA	Primer Bank	N/A
qPCR Primer: alpha-SMA Reverse: CCCCTGACAGGACGTTGTTA	Primer Bank	N/A
qPCR Primer: CDC20 Forward: GCCGAACTCCTGGCAAATCT	Primer Bank	N/A
qPCR Primer: CDC20 Reverse: TTGGGGGATAAAGCCGGTCAC	Primer Bank	N/A
qPCR Primer: CDK1 Forward: GGTCCGTCGTAACCTGTTGA	Primer Bank	N/A
qPCR Primer: CDK1 Reverse: CCACACCGTAAGTACCTTCTCC	Primer Bank	N/A
qPCR Primer: CDCA3 Forward: GAGGAAGCCAAACAATCCGC	Primer Bank	N/A
qPCR Primer: CDCA3 Reverse: TGCGCTTAGAACCTGAGGAC	Primer Bank	N/A
qPCR Primer: CENPA Forward: GGCCCTTCAGGAGACATTACC	Primer Bank	N/A
qPCR Primer: CENPA Reverse: GGCACCGTGTAGCCAGTATT	Primer Bank	N/A
qPCR Primer: Trp53 Forward: TCCGAAGACTGGATGACTGC	Primer Bank	N/A
qPCR Primer: Trp53 Reverse: GATCGTCCATGCAGTGAGGT	Primer Bank	N/A
qPCR Primer: CDKN1A Forward: GCAGAATAAAAGGTGCCACAGG	Primer Bank	N/A
qPCR Primer: CDKN1A Reverse: GACAACGGCACACTTTGCTC	Primer Bank	N/A
qPCR Primer: CDKN2A Forward: TGTGCATGACGTGCGGG	Primer Bank	N/A

qPCR Primer: CDKN2A Reverse: GCCCATCATCATCACCTGAATCG	Primer Bank	N/A
qPCR Primer: CCND1 Forward: CAGCCCCAACAACTTCCTCT	Primer Bank	N/A
qPCR Primer: CCND1 Reverse: CAGGGCCTTGACCGGG	Primer Bank	N/A
qPCR Primer: CDC7 Forward: TGGGATTTGGACATATGCTGCT	Primer Bank	N/A
qPCR Primer: CDC7 Reverse: AAGAGGCTTCCACTACGCAC	Primer Bank	N/A
qPCR Primer: BCL2 Forward: GACTGAGTACCTGAACCGGC	Primer Bank	N/A
qPCR Primer: BCL2 Reverse: AGTTCCACAAAGGCATCCCAG	Primer Bank	N/A
qPCR Primer: IRG1 Forward: ACTTCTCCAAGGAAGCCAAAGA	Primer Bank	N/A
qPCR Primer: IRG1 Reverse: ACTTTGTCAAGCTGAGCCCC	Primer Bank	N/A
qPCR Primer: TRIM21 Forward: CCCAGACCTAACAAGCACATG	Primer Bank	N/A
qPCR Primer: TRIM21 Reverse: TCTCCCATCCTCACTTGTCTC	Primer Bank	N/A
qPCR Primer: PSAT1 Forward: AAAAACAATGGAGGTGCCGC	Primer Bank	N/A
qPCR Primer: PSAT1 Reverse: GGCTCCACTGGACAAACGTA	Primer Bank	N/A
qPCR Primer: Gapdh Forward: CCTGGAGAAACCTGCCAAGTATG	Primer Bank	N/A
qPCR Primer: Gapdh Reverse: AGAGTGGGAGTTGCTGTTGAAGTC	Primer Bank	N/A
Chip-qPCR Primer: PSAT1-1 Forward: CAGCACTTCCCAGCAGACTG	Primer Bank	N/A
Chip-qPCR Primer: PSAT1-1 Reverse: GGAAGCTGTAGCGAAGGTGT	Primer Bank	N/A
Chip-qPCR Primer: PSAT1-2 Forward: GCGCGCTGTCTCTTCTGTTC	Primer Bank	N/A
Chip-qPCR Primer: PSAT1-2 Reverse: GGCCGGAGTAGGGAGACATC	Primer Bank	N/A
siRNAs		
PSAT1 siRNA (mus) Forward: CACUUAUGGAGGUGAUAUATT	HANBIO (Shanghai, China)	N/A
PSAT1 siRNA (mus) Reverse: UUAAGAUAUCCAUACCUCGTT	HANBIO (Shanghai, China)	N/A

ATF4 siRNA (mus) Forward: GCGAUCGAAUGUCAAAUAUTT	HANBIO (Shanghai, China)	N/A
ATF4 siRNA (mus) Reverse: AUAUUUGACAUUCGAUCGCTT	HANBIO (Shanghai, China)	N/A
IRG1 siRNA (mus) Forward: GAGGAUGAUUCUAGACACUTT	HANBIO (Shanghai, China)	N/A
IRG1 siRNA (mus) Reverse: AUGCUCUAGAAUCAUCCUCTT	HANBIO (Shanghai, China)	N/A
TRIM21 siRNA (mus) Forward: AGAUUCUUGAGCAGAAAGCGC	HANBIO (Shanghai, China)	N/A
TRIM21 siRNA (mus) Reverse: GCGCUUUCUGCUCAAGAAUCU	HANBIO (Shanghai, China)	N/A
Recombinant DNA		
Plasmid: PSAT1	HANBIO (Shanghai, China)	N/A
Plasmid: HSPA8-WT	HANBIO (Shanghai, China)	N/A
Plasmid: HSPA8-MUT	HANBIO (Shanghai, China)	N/A
Software and algorithms		
Bio-Rad Real-Time PCR System	Bio-Rad	N/A
Bio-Rad ChemiDoc MP System	Bio-Rad	N/A
Leica SP8 confocal microscope	Leica	N/A
FlowJo v10	FlowJo	N/A
Image J	N/A	https://imagej.nih.gov/ij/
Prism 7 and 8	GraphPad Software, Inc	N/A
